# Chemical proteasome inhibition as a novel animal model of inner retinal degeneration in rats

**DOI:** 10.1371/journal.pone.0217945

**Published:** 2019-05-31

**Authors:** Masaaki Kageyama, Takashi Ota, Masaaki Sasaoka, Osamu Katsuta, Katsuhiko Shinomiya

**Affiliations:** 1 Global Alliances and External Research, Santen Pharmaceutical Co., Ltd., Nara, Japan; 2 Research and Development Center, Santen Pharmaceutical Co., Ltd., Nara, Japan; Waseda University, JAPAN

## Abstract

Chemical proteasome inhibition has been a valuable animal model of neurodegeneration to uncover roles for the ubiquitin-proteasome system in the central nervous system. However, little is known about the effects of chemical proteasome inhibitors on retinal integrity. Therefore, we characterized the effects of structurally different chemical proteasome inhibitors on the retinal morphology and the mechanisms of their action in the normal adult rat eyes. Intravitreal injection of MG-262 and other proteasome inhibitors led to inner retinal degeneration. MG-262-induced inner retinal degeneration was accompanied by reduced proteasome activity, increased poly-ubiquitinated protein levels, and increased positive immunostaining of ubiquitin, 20S proteasome subunit and GADD153/CHOP in the retina. Its retinal degenerative effect was also associated with reduced retinal neurofilament light chain gene expression, reflecting retinal ganglion cell death. MG-262-induced neurofilament light chain downregulation was largely resistant to pharmacological modulation including endoplasmic reticulum stress, apoptosis or MAP kinase inhibitors. Thus, this study provides further evidence of roles for the ubiquitin-proteasome system in the maintenance of the retinal structural integrity. Chemical proteasome inhibition may be used as a novel animal model of inner retinal degeneration, including retinal ganglion cell loss, which warrants further analysis of the molecular mechanisms underlying its retinal degenerative effect.

## Introduction

The ubiquitin-proteasome system (UPS) is a crucial component of protein degradation processes, which contributes to protein quality control and proteome homeostasis in eukaryotic cells [1]. UPS-dependent protein degradation is regulated by three sequential enzymatic reactions, namely, 1) an adenosine-triphosphate (ATP)-dependent activation of ubiquitin by the ubiquitin activating enzyme E1, 2) ubiquitin ligation of the target protein by the conjugating enzyme E2, and the protein-ubiquitin ligase E3, and 3) proteolytic degradation of the tagged protein by the 26S proteasome complex. The 26S proteasome is composed of two 19S regulatory particles capping the 20S core particle that is a major catalytic site of ubiquitin-dependent protein degradation. The 20S proteasome also mediates ubiquitin-independent degradation of unfolded proteins subjected to oxidative stress [2]. UPS function declines with cellular senescence and aging in various organs and tissues including the central nervous system [1]. Furthermore, UPS dysfunction is associated with accumulation of misfolded and/or damaged proteins in the brain, which is the hallmark of several age-related neurodegenerative diseases such as Alzheimer, Parkinson and Huntington diseases [3]. Similarly, in the retina, proteasome activity declines in an age-dependent manner [4, 5]. Recent studies demonstrated that impaired proteasome function or proteasome overload caused retinal degeneration [6, 7], whereas increased proteasome activity prevented it [8]. These results suggest that UPS dysfunction is also associated with age-related and inherited retinal degenerative diseases caused by misfolded and damaged proteins. Thus, the UPS can be a potential therapeutic target for these retinal diseases.

Several chemical proteasome inhibitors have been developed and prescribed for the treatment of patients with multiple myeloma [9]. Simultaneously, they have been used as an indispensable research tool for uncovering its biological roles in neurodegeneration. Earlier studies reported that neurodegeneration induced by intracerebral [10–12] or systemic administration [10, 13] of chemical proteasome inhibitors in rodents recapitulated the key clinical features of Parkinson’s disease, namely, dopaminergic neuronal death and impaired motor coordination, which were accompanied by formation of inclusion bodies enclosing aggregated α-synuclein. These studies have established chemical proteasome inhibition-induced neurodegeneration as a simple and valuable animal model for further UPS research as well as drug discovery [10]. Despite such progress in research on Parkinson’s disease, no attempt has yet been made to create and characterize an *in vivo* model of retinal degeneration using chemical proteasome inhibition.

The present study was therefore designed to examine the effects of structurally different chemical proteasome inhibitors on the retinal morphology. Here, we report, for the first time, that intravitreal injection of chemical proteasome inhibitors leads to inner retinal degeneration, including retinal ganglion cell (RGC) death, in the normal adult rat retina. We also demonstrate that proteasome inhibition-induced retinal degeneration is accompanied by reduced proteasome activity and increased poly-ubiquitinated protein levels in the retina.

## Materials and methods

### Chemicals

MG-262 and lactacystin were purchased from Wako Pure Chemical Industries, Ltd. (Osaka, Japan) and Peptide Institute Inc (Osaka, Japan), respectively, whereas bortezomib and 17-DMAG were purchased from LC laboratories (MA, USA). N-acetyl cysteine (NAC), U0126 and sodium 4-phenyl butyrate (PBA, Wako Pure Chemical Industries, Ltd., Osaka, Japan), memantine (Sequoia, Pangbourne, UK), lomerizine (Organon, Roseland, NJ, USA), nafamostat (Yick-Vic Chemicals & Pharmaceuticals, Hong Kong), salubrinal and KB-R7943 (Tocris, Avenmouth, Bristol, UK), Z-VAD-FMK (ZVAD), SB-216763 and ansatrienin A (Enzo Life Sciences, Farmingdale, NY), UBEI-41 (4 [4-(5-Nitro-furan-2-ylmethylene)-3,5-dioxo-pyrazolidin-1-yl]-benzoic acid ethyl ester, Biogenova, Potomac, MD), aldehyde dehydrogenase (ALDH, Fluka, Mexico city, Mexico), and brain-derived neurotrophic factor (BDNF, Millipore, Darmstadt, Germany) were provided by the respective chemical vendors. Thapsigargin, AS601245, aurintricarboxylic acid, ZVAD, pifithrin-α, cyclic (pifithrin), TWS119, bax channel blocker ((±)-1-(3,6-Dibromocarbazol-9-yl)-3-piperazin-1-yl-propan-2-ol, bis TFA), C2-8 (N-(4-bromophenyl)-3-[[(4-bromophenyl)amino]sulfonyl]benzamide; N-(4-bromophenyl)-3-[(4-bromophenyl)sulfamoyl]benzamide), and LDN-57444 ([(Z)-[5-chloro-1-[(2,5-dichlorophenyl)methyl]-2-oxoindol-3-ylidene]amino] acetate) were purchased from Calbiochem (La Jolla, CA). All other chemicals were obtained from Sigma (St Louis, MO). All chemicals were dissolved in distilled water or DMSO to prepare a stock solution and diluted with Dulbecco’s phosphate buffered solution (D-PBS) or distilled water to obtain a given final concentration.

### Animals and intravitreal injection

All animals were treated in compliance with the ARVO statement for the Use of Animals in Ophthalmic and Vision Research. The experimental procedure was approved and monitored by the Animal Care and Use Committee of the Nara Research & Development Center, Santen Pharmaceutical Co., Ltd. Male Sprague Dawley rats were purchased from Japan SLC Inc (Shizuoka, Japan), housed under a 12-h light/12-h dark cycle and provided food and water ad libitum. Rats weighing 190 to 270g were anesthetized with inhalation of 3–4% isoflurane and maintained with 1–2% isoflurane. Following pupil dilatation with topical application of tropicamide and phenylephrine hydrochloride (Mydrin-P, Santen Pharmaceutical Co., Ltd., Osaka, Japan), a 5 μl aliquot of solution containing each proteasome inhibitor was injected into the vitreous body of both eyes of each animal through a Hamilton microsyringe with a 33G needle. For concomitant injection of a proteasome inhibitor with any of other chemicals, both chemicals were premixed and a 5 μl aliquot of resultant solution was administered in the same way as described above. All injections were performed under a microscope used for ocular surgery and care was taken not to injure the lens or retina during the procedure. A bilateral approach was taken to minimize the number of animals sacrificed in this study, as seen in earlier studies [14, 15]. One, three or seven days following injections, animals were euthanized with intraperitoneal injection of excess dose of pentobarbital. The eyes were enucleated and processed for further analysis as described in each section below.

### Histological evaluation

The isolated eyes were fixed in a neutral buffered solution containing 10% formaldehyde and 2.5% glutaraldehyde for 24 hrs at room temperature. The fixed eyes were embedded in paraffin, cross sectioned and stained with hematoxylin and eosin. Eight cross sections (3 μm thickness) of the retina through the optic disk at 45 μm intervals were prepared and three out of eight sections were randomly chosen for histological evaluation. The light microscopic images of the retina were captured with a fully automated digital slide scanner (NanoZoomer Digital Pathology, Hamamatsu Photonics, Shizuoka, Japan). Each image included approximately the 800 μm width of the retina, which started at a distance of 700 μm from the center of the optic disk. For each image, the number of cells in the ganglion cell layer (GCL) and the thickness of inner plexiform layer (IPL) were determined. The averaged value among three sections was used as the representative value for each eye.

### Proteasome activity assay

The *in vitro* activities of proteasome were determined in a cell free assay system according to the manufacturer’s instruction manual (Biomol, Plymouth Meeting, PA). Namely, a 5-μl aliquot of each proteasome inhibitor solution was added to 45 μl reaction buffer containing 0.2 μg 20S proteasome, 1% SDS and 75 μM Suc-Leu-Leu-Val-Tyr-7-amino-4-methylcoumarin (Suc-LLVY-AMC) and incubated at room temperature in a 96-well microplate. Fluorescence signal was monitored over time at the wavelengths of 360 and 460 nm by a fluorescence microplate reader (CytoFluor Multi-well plate reader Series 4000, Applied Biosystems, Framingham, MA). IC_50_ was determined by linear regression analysis for each of concentration-response curves. For *ex vivo* proteasome activity measurements, the retinae were isolated and frozen on dry ice immediately following enucleation of the eyes. Each retina was homogenized in a 20 mM Tris-acetate buffered solution (pH7.2) containing 20% sucrose, 2 mM MgCl_2_, 10 mM glucose and 0.05% Nonidet P40. Retinal proteasome activities were determined according to the manufacturer’s instruction manual (Calbiochem, San Diego, CA). Briefly, a 35-μl aliquot of the supernatant was added to 165 μl reaction buffer containing 1% SDS and 10 μM Suc-LLVY-AMC in a 96-well microplate and fluorescence signal was monitored as described above. For each retinal extract, a protein concentration was also determined by a BCA protein assay kit (Pierce Biotechnology, Rockford, IL). Retinal proteasome activity was normalized to a total protein content in each retinal sample.

### ELISA for determination of poly-ubiquitinated protein

The isolated retinae were frozen on dry ice and homogenized in a 200-μl cell extraction buffer containing protease inhibitor cocktail. Poly-ubiquitinated protein contents were determined by a solid phase sandwich ELISA using anti-poly-ubiquitin monoclonal antibody according to the manufacturer’s instruction manual (CycLex, Nagano, Japan). Briefly, a 100-μl aliquot of diluted supernatant or a serial dilution of standard solution was added to the wells of a 96-well plate pre-coated with anti-poly-ubiquitinated protein antibody and incubated at room temperature for an hour. The wells were washed and further incubated with a 100-μl aliquot of HRP-conjugated antibody solution for an hour. Tetra-methylbenzidine was used for a chromogenic reaction and absorbance at the wavelength of 450 nm was measured by a spectrophotometric microplate reader (Model 3550, Biorad, Hercules, CA). A poly-ubiquitinated protein content was normalized to a total protein content in each retinal sample, which was determined by a BCA protein assay kit.

### Immunohistochemistry

The isolated eyes were fixed in a 0.1M phosphate-buffered solution containing 4% paraformaldehyde for 24 hrs at 4°C. The fixed eyes were immersed in ascending concentrations of sucrose from 7 up to 30%, embedded in OTC compound and rapidly frozen on ice with isopentane. Serial cross sections (4-μm thickness) of the retina through the optic disk were prepared and blocked with 2% bovine serum albumin in D-PBS at room temperature for at least 30 min following endogenous peroxidase blocking. The retinal sections were incubated at 4°C for 16–18 hours with either primary antibody against ubiquitin (1:200, rabbit polyclonal, Cat No. Z0458, Agilent Dako, Santa Clara, CA), 20S proteasome core subunits (1:1000, rabbit polyclonal, Cat No. PW8155, Enzo Life Sciences, Farmingdale, NY) or GADD153/CHOP (CHOP, 1:50, rabbit polyclonal, Cat No. sc-575, SantaCruz, Santa Cruz, CA). The sections were subsequently washed with D-PBS and incubated with a Fab-protein complex labelled with peroxidase (Simple stain MAX PO MULTI, Nichirei, Tokyo, Japan) at room temperature for 60 min. For negative controls, a primary antibody was replaced by mouse IgG1 or rabbit immunoglobulin fraction (Agilent Dako, Santa Clara, CA). The sections were stained with diaminobenzidine and counterstained with hematoxylin.

### Real time PCR

The retinae were isolated and stored at -20°C in RNA later (Qiagen, Hilden, Germany) until the day of RNA extraction. Total RNA was individually extracted from each retina using an RNeasy 96 kit (Qiagen, Hilden, Germany) according to the manufacturer’s instructions. Namely, the retina was homogenized in a 2 ml tube containing 0.5 ml QIAzol and a single zirconia bead by Tissue Lyzer (Qiagen, Hilden, Germany). A 100 μl aliquot of chloroform was added to the homogenate and the mixture was centrifuged at 12,000g at 4°C for 5 min. The supernatant was transferred to a new tube and the same amount of 70% ethanol was added. The mixture was applied onto the wells in an RNase 96 plate and total RNA was eventually eluted with RNase-free water into a regular 96-well plate. First strand cDNA was prepared using 0.1 μg of total RNA for each retina in a 20 μl mixture containing 4 μl of PrimeScript buffer, 1 μl of PrimeScript RT Enzyme Mix ǀ, 25 pmol oligo-dT primers and 50 pmol random 6 mers (Takara, Shiga, Japan). An aliquot of resultant cDNA was added to a reaction mixture of 10 μl QuantiTect Multiplex PCR Master Mix (Qiagen, Hilden, Germany), 1 μl pre-designed primer-probe mixture for GAPDH (Applied Byosystems, Foster City, CA) and neurofilament light chain (NFL, Sigma-Aldrich, St. Louis, MO), and 5 μl RNase free water. For NFL, the sequences of forward and reverse primers used were 5’-ACAAGCAGAATGCAGACATCA-3’ and 5’-GGAGGTCCTGGTACTCCTTC-3’, respectively, whereas the sequence of TaqMan probe was [FAM] 5’- CCATCTCGCTCTTCGTGCTTCGC -3’ [BHQ-1]. The sequences of primers and probe for GAPDH are kept undisclosed by the manufacturer. Real time PCR was performed using a 7500 Fast Real-Time PCR system (Applied Byosystems, Foster City, CA) under the cycling conditions of 50°C for 2 min and 95°C for 15 min followed by 40 cycles of 94°C for 1 min and 60°C for 1 min. For some samples, real time PCR was performed with a QuatiFast kit according to the manufacturer’s instruction manual (Qiagen, Hilden, Germany). Fluorescence intensity was measured at every annealing step and was analyzed using the 7500 software to obtain threshold cycle time (C_T_) values. Gene expression level of NFL relative to that of GAPDH was determined according to the comparative C_T_ method and further normalized to the respective control (untreated or vehicle group).

### Statistical analysis

Each value represents the mean ± S.E.M. For statistical analysis, one-way analysis of variance (ANOVA) was performed and followed by post-hoc analysis, if significant. Dunnett’s multiple comparison test was used for comparison between the control and each treatment group, whereas Tukey’s test was used for comparison among all groups. Differences were assumed to be statistically significant when *P*<0.05.

## Results

### Retinal degeneration following intravitreal injection of chemical proteasome inhibitors and endoplasmic reticulum (ER) stress inducers

**Fig 1:**
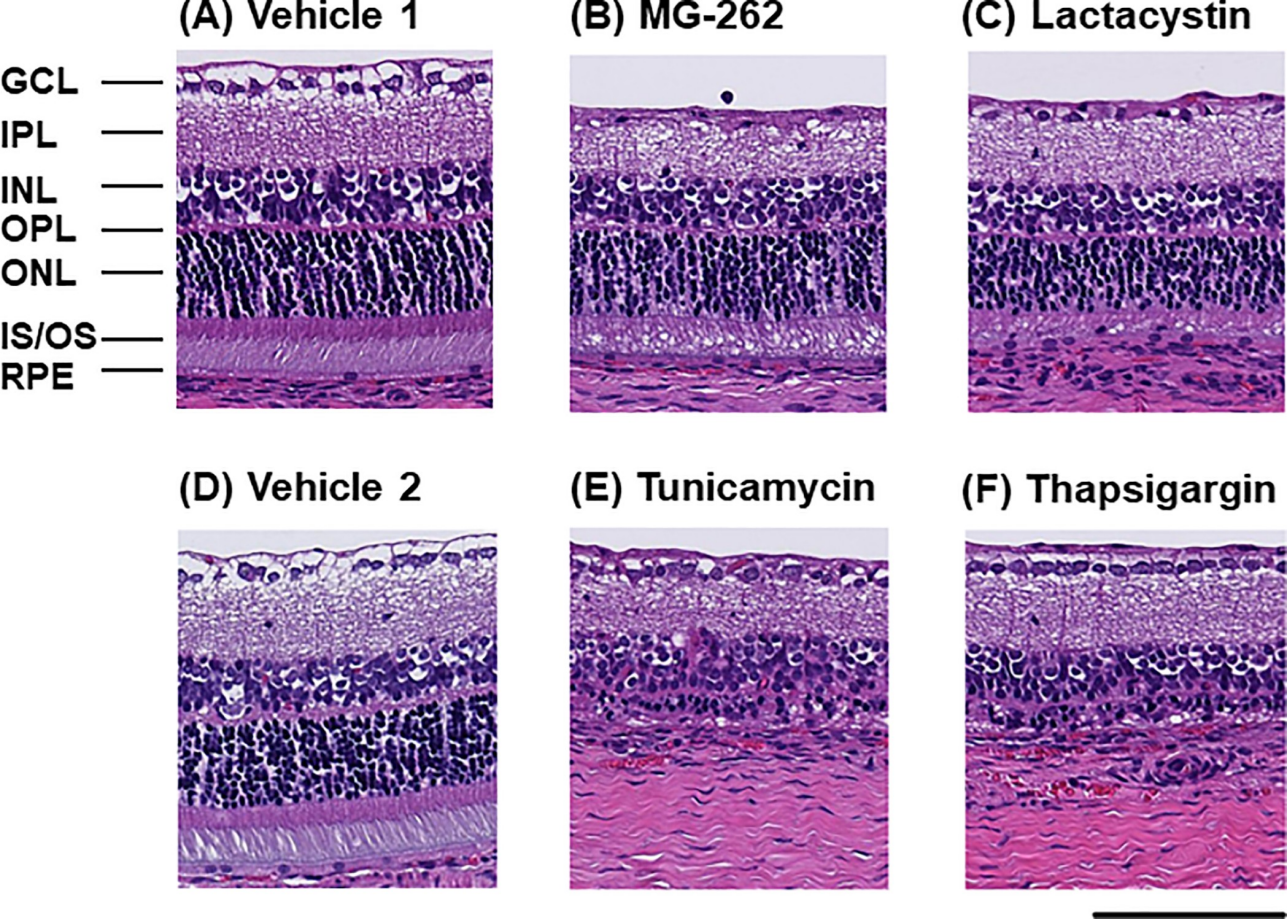
Retinal degeneration induced by MG-262, lactacystin, tunicamycin and thapsigargin in the normal adult rat eyes. Each photograph shows the representative image of cross-sectioned retina 7 days following intravitreal injection of vehicle (A and D, 10% DMSO in D-PBS), MG-262 (B, 0.27 nmol/eye), lactacystin (C, 10 nmol/eye), tunicamycin (E, 2.5 nmol/eye), and thapsigargin (F, 10 nmol/eye). The scale bar shows 100 **μ**m. GCL: ganglion cell layer; IPL: inner plexiform layer; INL: inner nuclear layer; OPL: outer plexiform layer; ONL: outer nuclear layer; IS/OS: photoreceptor inner and outer segments; RPE: retinal pigment epithelium.

First, we examined the effects of 20S proteasome inhibitors, MG-262 [16] and lactacystin [17], on the retinal morphology in comparison with those of the ER stress inducers, tunicamycin and thapsigargin [18]. This is because we assumed that accumulation of misfolded proteins following chemical proteasome inhibition would lead to ER stress responses, as seen with these ER stress inducers. Fig 1A–1F show the representative images of the retinal morphology following intravitreal injection of proteasome inhibitors and ER stress inducers. Seven days following intravitreal injection, MG-262 (0.27 nmol/eye, Fig 1B) caused severe loss of cells in GCL accompanied by the slightly thinner IPL and vacuoles in the photoreceptor inner and outer segments (IS and OS) compared with the vehicle control (Fig 1A). The inner nuclear layer (INL) and the outer nuclear layer (ONL) were relatively preserved and appeared to be normal. However, electron microscopic observation of the retina exposed to MG-262 (0.1 nmol/eye) revealed nuclear condensation and/or necrotic damage in both layers 24 hours following injection (see S1 Fig). Intravitreal injection of lactacystin (10 nmol/eye) caused a very similar pattern of retinal degeneration represented by cell loss in GCL (Fig 1C). In contrast, intravitreal injection of tunicamycin (2.5 **μ**g/eye) and thapsigargin (10 nmol/eye) led to massive photoreceptor degeneration with complete loss of ONL, IS and OS (Fig 1E and 1F) compared with the other vehicle control (Fig 1D). Interestingly, the inner retina was well-preserved and, particularly, the cell number in GCL was not different from that in the vehicle control group. Thus, their distinct retinal degenerative patterns suggest that the mechanisms of action underlying retinal degeneration induced by chemical proteasome inhibitors and ER stress inducers are not identical.

**Fig 2:**
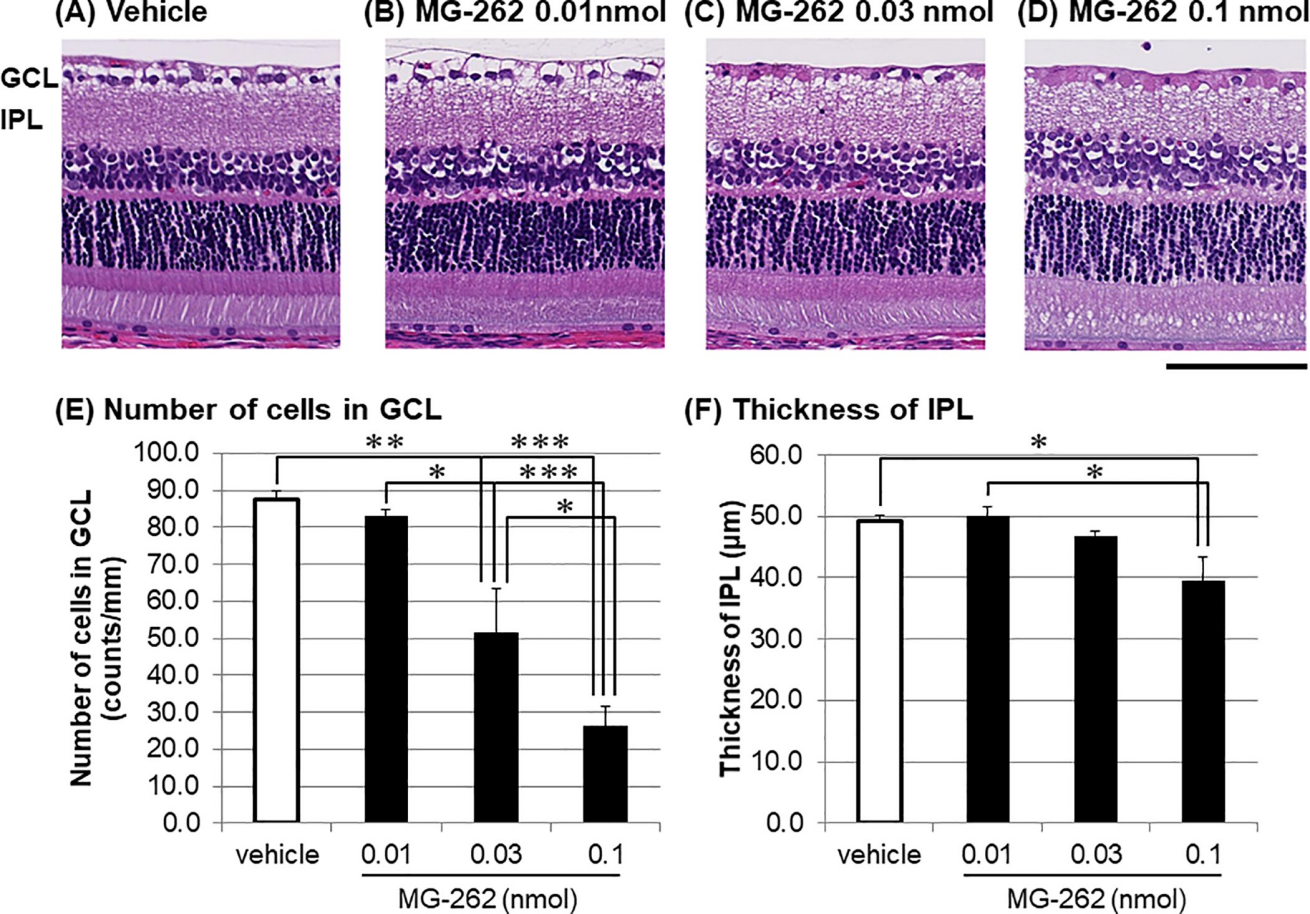
Dose-dependency of retinal degeneration induced by MG-262 in the normal adult rat eyes. Each photograph shows the representative image of cross-sectioned retina 7 days following intravitreal injection of vehicle (A, 10% DMSO in D-PBS) and various concentrations of MG-262 (B: 0.01 nmol/eye; C: 0.03 nmol/eye; D: 0.1 nmol/eye). The scale bar shows 100 μm. The lower left and right panels show the number of cells in the ganglion cell layer (GCL, E) and the thickness of the inner plexiform layer (IPL, F), respectively. Each value represents the mean ± S.E.M. of 3 to 4 eyes from 2 to 3 animals. *P<0.05; **P<0.01; ***P<0.001, by Tukey’s multiple comparison test.

We further characterized dose-dependency of the retinal degenerative effect of MG-262. Seven days following intravitreal injection, MG-262 demonstrated dose-dependent loss of cells in GCL at the doses ranging from 0.01 to 0.1 nmol/eye (Fig 2B–2D) compared with the vehicle control (Fig 2A). The changes in the cell number in GCL were statistically significant at the middle and highest doses (Fig 2E). In addition, the differences among all three doses reached statistical significance. The thickness of IPL was also reduced following MG262 injection in a dose-dependent manner (Fig 2A–2D), but the degree of changes was smaller than that in the cell number in GCL (Fig 2E and 2F). The differences were statistically significant between vehicle and the highest dose, and between the lowest and highest doses (Fig 2F). At the lowest and middle doses, the outer retina appeared to be normal, whereas vacuoles in IS and OS were observed at the highest dose (Fig 2B–2D).

### Retinal degeneration induced by intravitreal injection of other UPS-related inhibitors

**Fig 3:**
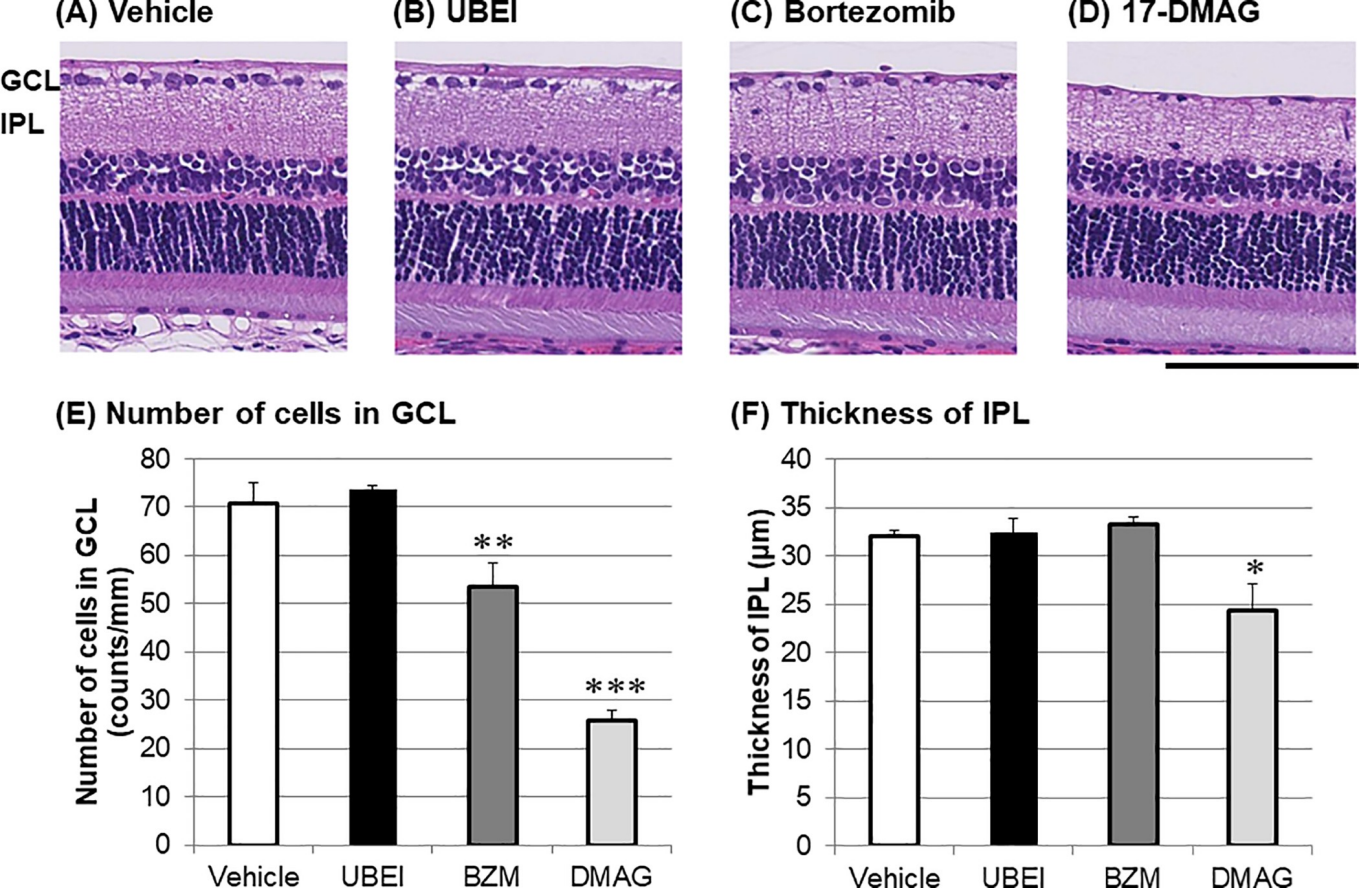
Retinal degeneration induced by bortezomib (BZM) and 17-DMAG (DMAG) in the normal adult rat eyes. Each photograph shows the representative image of cross-sectioned retina 7 days following intravitreal injection of vehicle (A, D-PBS), UBEI-41 (UBEI, B: 12.5 nmol/eye), BZM (C: 1 nmol/eye), and DMAG (D: 25 nmol/eye). The scale bar shows 100 μm. The lower left and right panels show the number of cells in the ganglion cell layer (GCL, E) and the thickness of the inner plexiform layer (IPL, F), respectively. Each value represents the mean ± S.E.M. of 4 eyes from 2 animals. *P<0.05; **P<0.01; ***P<0.001, compared with vehicle by Dunnett’s multiple comparison test.

To extend our understanding of chemical proteasome inhibition-induced retinal degeneration, we examined the effects of other UPS-related inhibitors on the retinal morphology. UBEI-41 [19] and LDN-57444 [20] are inhibitors for E1 enzyme and UCH-L1, which catalyze ubiquitin activation and deubiquitination in the UPS, respectively. Seven days following intravitreal injection, either UBEI-41 (12.5 nmol/eye, Fig 3B, 3E and 3F) or LDN-57444 (2.5 nmol/eye, see S2 Fig) had no effect on the cell number in GCL or the thickness of IPL. In contrast, intravitreal injection of bortezomib (1 nmol/eye), another 20S proteasome inhibitor [21], led to statistically significant cell loss in GCL (Fig 3C and 3E), as seen with MG-262 and lactacystin. We further examined the effects of two additional inhibitors, the HSP90 inhibitor 17-DMAG [22] and the protein translocation inhibitor brefeldin A [23], on the retinal morphology. 17-DMAG (25 nmol/eye) caused statistically significant loss of cells in GCL accompanied by the slightly reduced thickness of IPL 7 days following intravitreal injection (Fig 3D, 3E and 3F), both of which were very similar to the changes observed with other proteasome inhibitors. Brefeldin A (1.5 nmol/eye) had no effect on either the cell number in GCL or the thickness of IPL (see S2 Fig). In the UPS-mediated protein degradation machinery, 20S proteasome may be the most important component to mediate chemical proteasome inhibition-induced inner retinal degeneration.

### Inhibitory effects of various inhibitors on 20s proteasome activity in vitro and ex vivo

**Fig 4:**
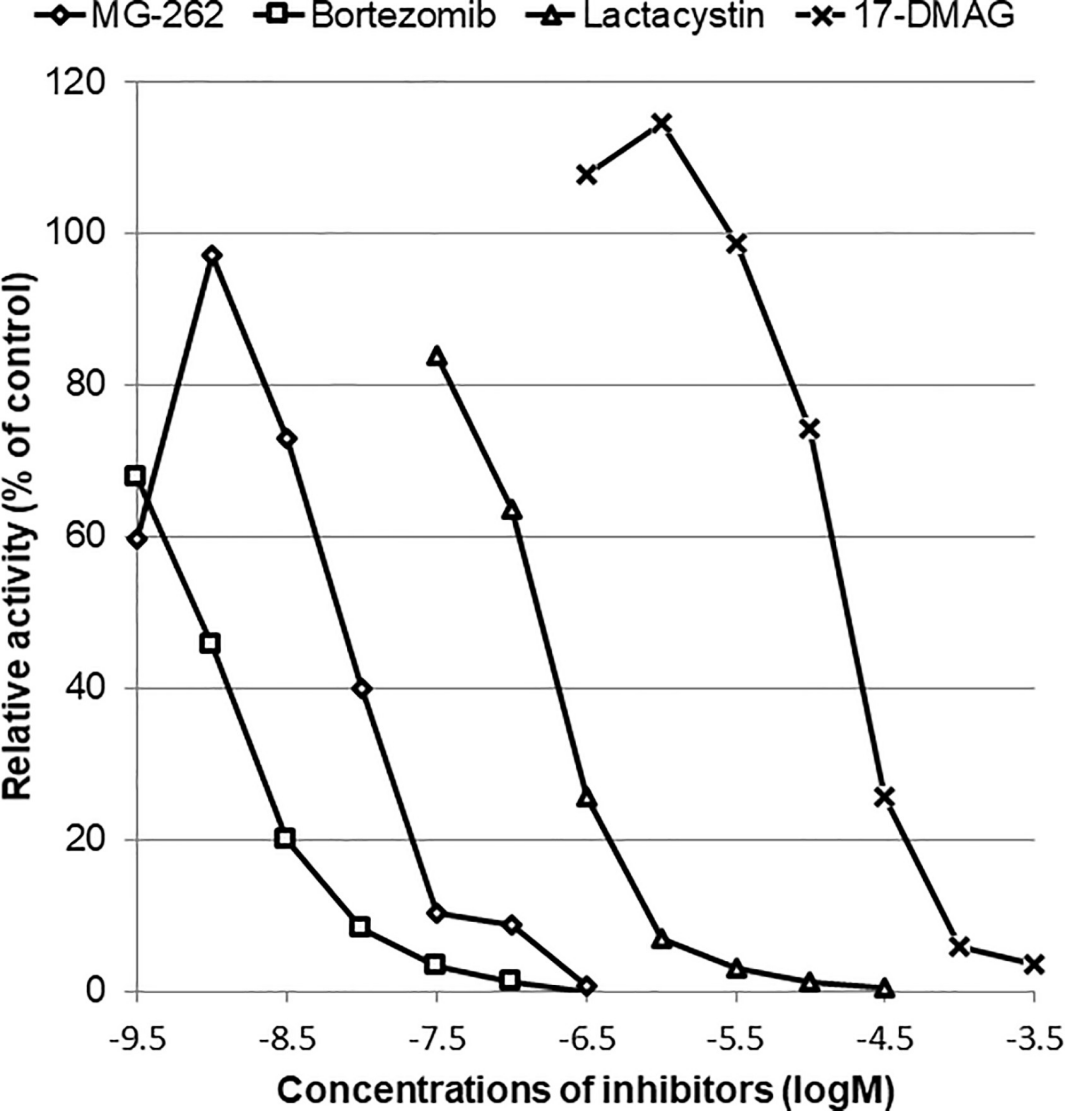
Concentration-response curves for inhibitory effects of MG-262 (open diamond), bortezomib (open square), lactacystin (open triangle) and 17-DMAG (cross) on proteasome activity in a cell-free assay. Proteasome activities were determined by a fluorometric method in the presence or absence of each inhibitor and normalized to the control value in the absence of any inhibitor. The relative activity is shown as percentage of the control. Each value represents the mean of duplicate measurements.

**Fig 5:**
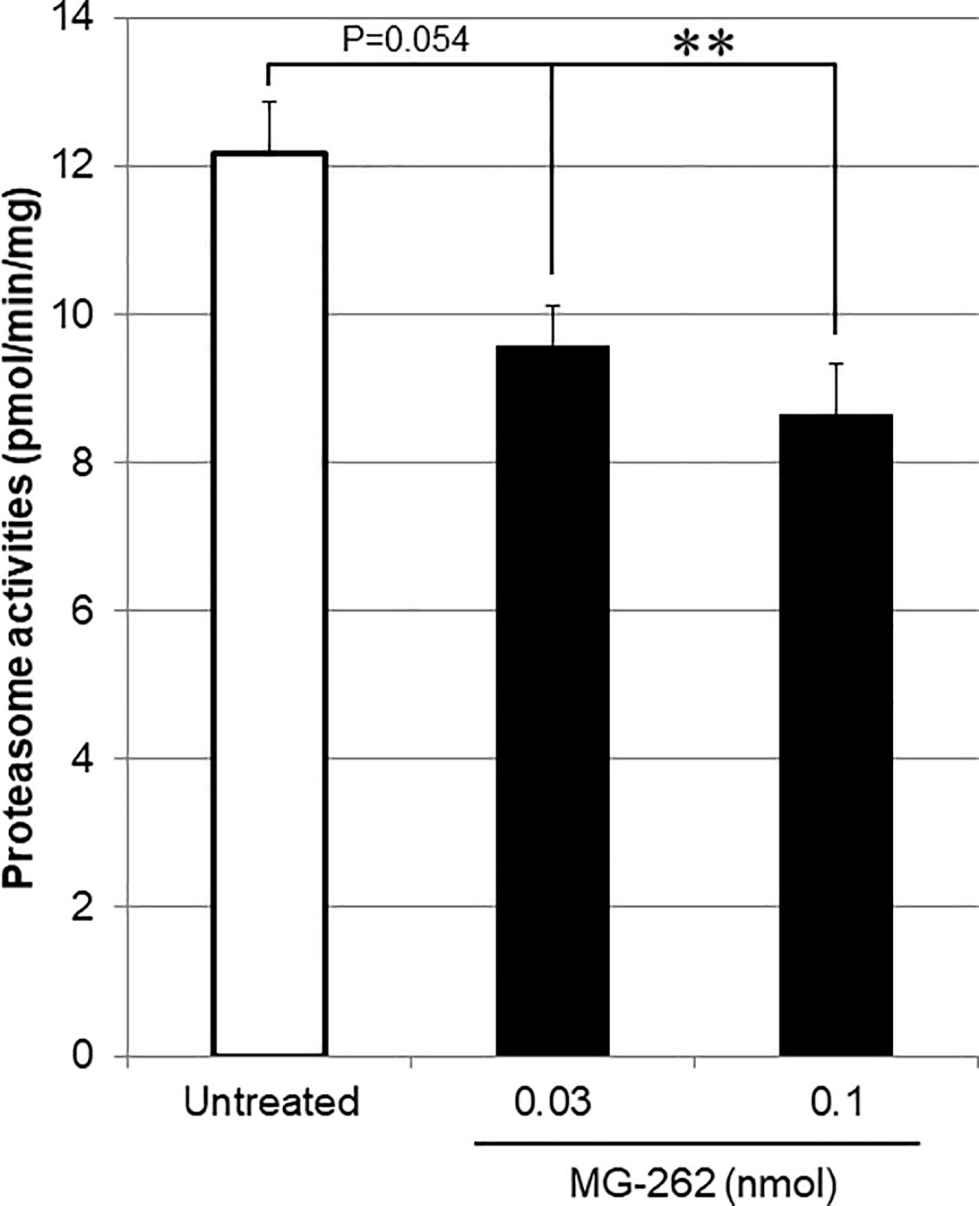
Inhibitory effects of MG-262 on retinal proteasome activity *ex vivo*. MG-262 (closed columns) was intravitreally injected at the doses of 0.03 and 0.1 (nmol/eye). For the control group (open column), the eyes were left untreated. Three days following intravitreal injection, the retina was isolated and proteasome activity in the retinal lysate was determined by a fluorometric method. Retinal proteasome activity was normalized to a total protein content in each retinal lysate. Each value represents the mean ± S.E.M. of 4 to 5 eyes from 2 to 3 animals. **P<0.01, by Tukey’s multiple comparison test.

Next, we determined using cell-free and *ex vivo* enzymatic assays whether the retinal degenerative effects of chemical proteasome inhibitors would be associated with reduced proteasome activity in the retina. As shown in Fig 4, MG-262, bortezomib, and lactacystin reduced proteasome activity in a concentration-dependent manner in a cell free assay. The rank order of their inhibitory effects on proteasome activity was bortezomib > MG-262 > lactacystin with IC_50_ of -9.12, -8.16, and -6.84 (logM), respectively. Unexpectedly, 17-DMAG reduced proteasome activity with IC50 of -4.78 (logM) in the same assay, although its inhibitory effect was much weaker than those of any other proteasome inhibitors used in this study. *Ex vivo* retinal proteasome activity was also determined 3 days following intravitreal injection of MG-262 at 0.03 and 0.1 nmol/eye (Fig 5). The inhibitory effects of MG-262 on *ex vivo* retinal proteasome activity were dose-related, and the changes at the higher dose reached statistical significance.

### Poly-ubiquitinated protein accumulation following intravitreal injection of MG-262

**Fig 6:**
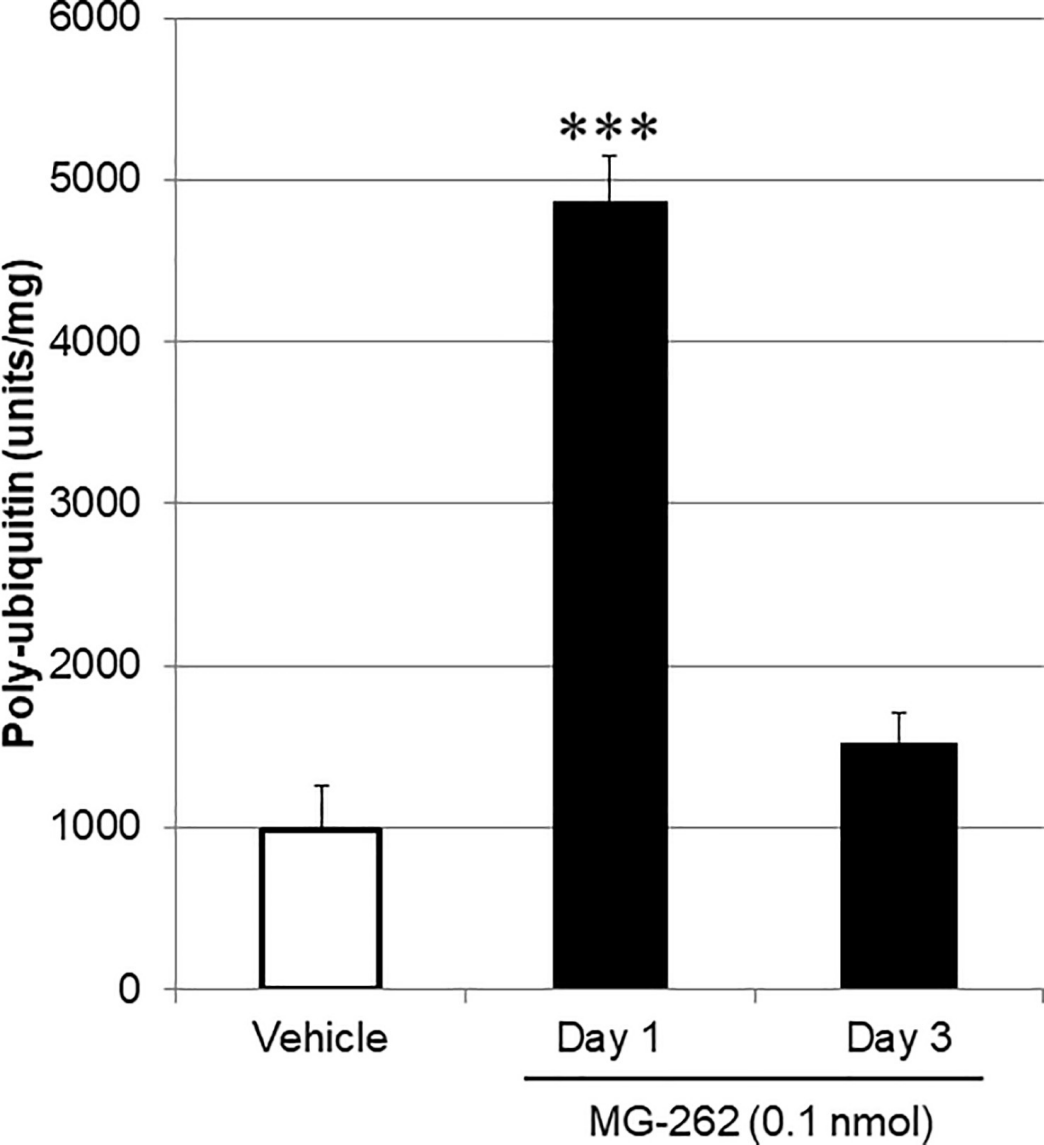
Increased poly-ubiquitinated protein levels in the retina following intravitreal injection of MG-262. MG-262 (closed columns) was intravitreally injected at the dose of 0.1 (nmol/eye). For the control group (open column), vehicle (10% DMSO in distilled water) was injected. One and three days following intravitreal injection, the retina was isolated and poly-ubiquitinated protein levels in each retinal lysate were determined by ELISA. The retinal poly-ubiquitinated protein level was normalized to a total protein content in each retinal lysate. Each value represents the mean ± S.E.M. of 4 eyes from 2 animals. ***P<0.001, compared with vehicle by Dunnett’s multiple comparison test.

**Fig 7:**
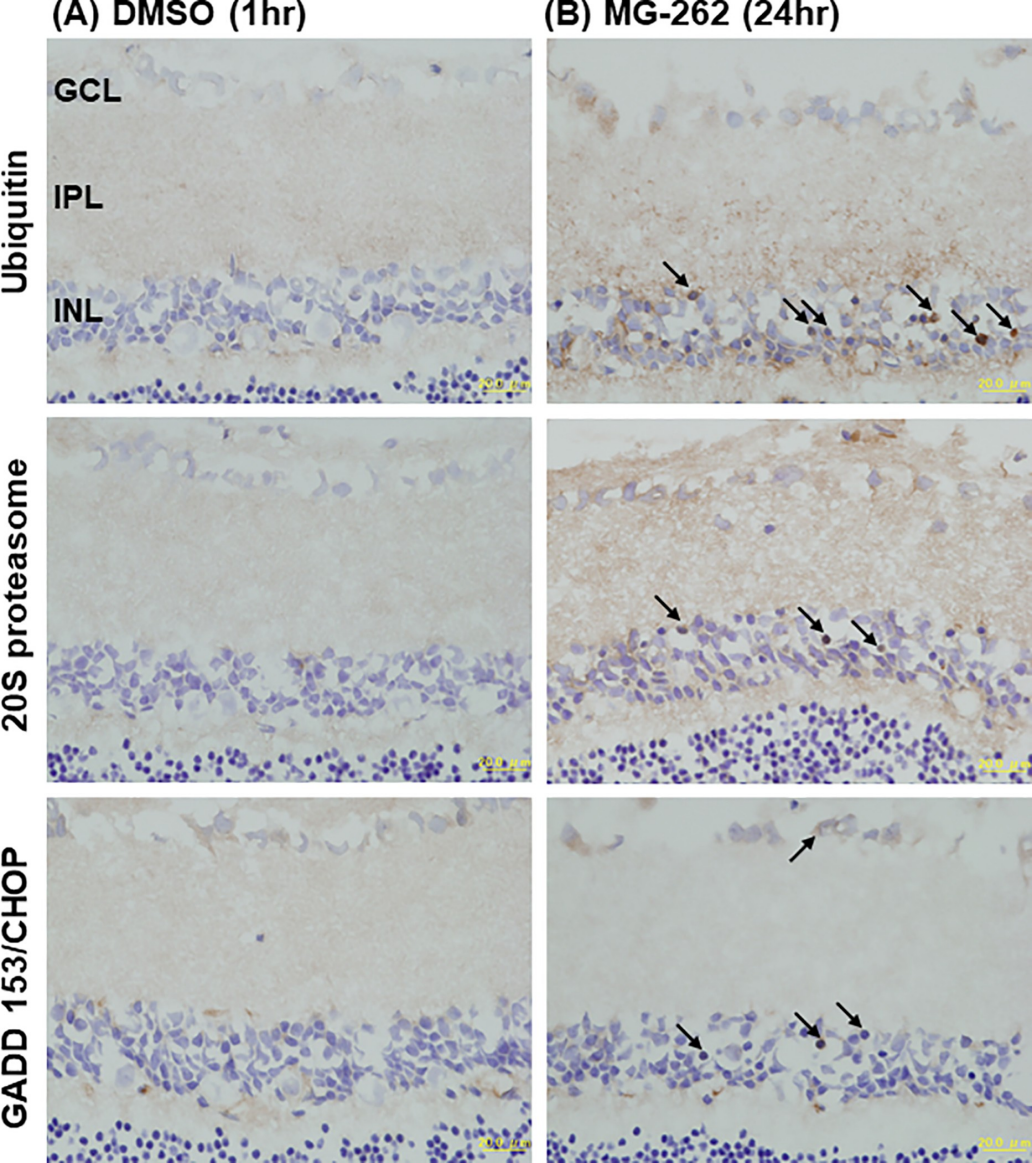
Increased ubiquitin-positive immunohistochemical staining in the retina following intravitreal injection of MG-262. Each photograph shows the representative image of cross-sectioned retina following intravitreal injection of vehicle (A, 50% DMSO in distilled water) and MG-262 (B, 0.1 nmol/eye). Eyes were enucleated 1 and 24 hrs following injection and the retina was subjected to immunohistochemical staining using antibodies against ubiquitin (upper), 20S proteasome subunit (middle) and GADD153/CHOP (lower). Arrows show representative cells positively stained with each antibody. Each scale bar shows 20 μm. GCL: ganglion cell layer; IPL: inner plexiform layer; INL: inner nuclear layer.

To further validate whether the retinal degenerative effect of MG-262 was mediated by inhibition of retinal proteasome activity, we took two different approaches, 1) quantification by ELISA of the poly-ubiquitinated protein levels in retinal extracts (Fig 6), and 2) qualitative characterization by immunohistochemistry of tissue localization of ubiquitin and the 20S proteasome subunit in the retina (Fig 7). Following intravitreal injection of MG-262 at 0.1 nmol/eye, the poly-ubiquitinated protein levels in the retina were remarkably increased on Day 1 and the change was statistically significant (Fig 6). Subsequently, the poly-ubiquitinated-protein levels dropped toward the baseline observed in the vehicle-treated group on day 3 following injection. Although the levels were numerically higher than the baseline, these changes were no longer statistically significant. No changes in the poly-ubiquitinated protein levels were observed 3 days following intravitreal injection of MG-262 at 0.03 nmol/eye (see S3 Fig).

Fig 7A and 7B show the representative immunohistochemical images of the retina stained with antibodies against ubiquitin, the 20S proteasome subunit and CHOP, which is an indicator of ER stress [24]. Following intravitreal injection of vehicle alone, only marginal signals for each of target proteins were observed throughout the retina at 1 hr (Fig 7A). MG-262 (0.1 nmol/eye) increased ubiquitin-positive immunochemical staining in IPL, INL, OPL, and ONL 24 hrs following injection (Fig 7B). Similarly, increased staining of 20S proteasome was observed in the same retinal layers following MG-262 injection, suggesting a compensatory mechanism for impaired proteasome function [13]. The increased ubiquitin- and 20S proteasome-positive staining was accompanied by increased CHOP-positive staining, but it was observed only in cell bodies in GCL and INL. No noticeable immunostaining was observed in the IS/OS or RPE layer (See S1 Table). Thus, both of increased retinal poly-ubiquitinated protein levels and ubiquitin-positive immunostaining by MG-262 are consistent with reduced retinal proteasome activity. Collectively, it is likely that impairment of retinal proteasome function by MG-262 and possibly other proteasome inhibitors may be responsible for inner retinal degeneration.

#### Pharmacological modulation of retinal degeneration induced by MG-262 and 17-DMAG

**Fig 8:**
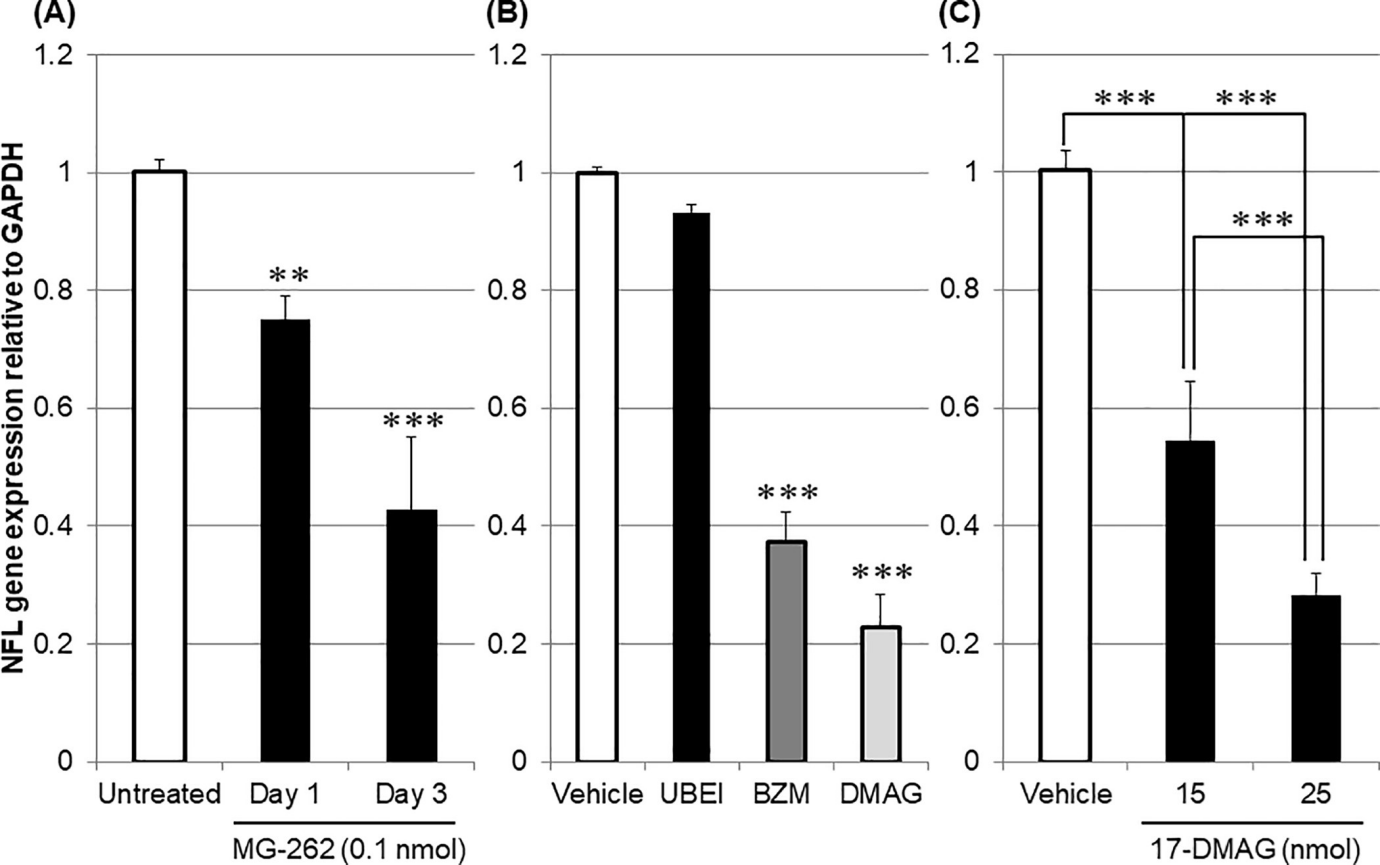
Downregulation of neurofilament light chain (NFL) gene expression following intravitreal injection of proteasome inhibitors. (A) Time course of NFL downregulation 1 and 3 days following MG-262 injection (closed column, 0.1 nmol/eye). The eyes in the control group were left untreated (open column). (B) NFL downregulation 3 days following injection of UBEI-41 (UBEI, closed column, 12.5 nmol/eye), bortezomib (BZM, dark-grey column, 1 nmol/eye) and 17-DMAG (DMAG, light-grey column, 25 nmol/eye). For the control group (open column), vehicle (D-PBS) was injected into the eyes. (C) Dose-dependency of NFL downregulation 1 day following 17-DMAG injection (15 and 25 nmol/eye, closed column). Vehicle (20% DMSO in distilled water) was injected into the eyes in the control group (open column). Following intravitreal injection, the retina was isolated and NFL gene expression was determined by real time PCR. The NFL gene expression level was normalized to that of GAPDH in each retinal sample and shown as the value relative to the respective control. Each value represents the mean ± S.E.M. of 4 to 7 eyes from 2 to 4 animals. **P<0.01; ***P<0.001, compared with the untreated and vehicle-treated groups by Dunnett’s multiple comparison test in (A) and (B), respectively. ***P<0.001, by Tukey’s multiple comparison test in (C).

To explore the molecular mechanisms underlying chemical proteasome inhibition-induced inner retinal degeneration, we examined the effects of a wide variety of pharmacological agents on RGC loss caused by MG-262 and 17-DMAG. In this experiment, NFL gene expression was used as a marker for the RGC number, as seen in earlier studies [25]. As shown in Fig 8A–8C, we observed remarkable downregulation of NFL gene expression following intravitreal injection of proteasome inhibitors. MG-262 (0.1 nmol/eye) downregulated NFL gene expression in a time-dependent manner on day 1 and 3 following injection (Fig 8A). Bortezomib (1 nmol/eye) and 17-DMAG (25 nmol/eye), but not UBEI-41 (12.5 nmol/eye), downregulated NFL gene expression on day 3 (Fig 8B). The downregulation of NFL gene expression by 17-DMAG (15 and 25 nmol/eye) on day 1 was dose-dependent (Fig 8C). All of these changes reached statistical significance.

**Fig 9:**
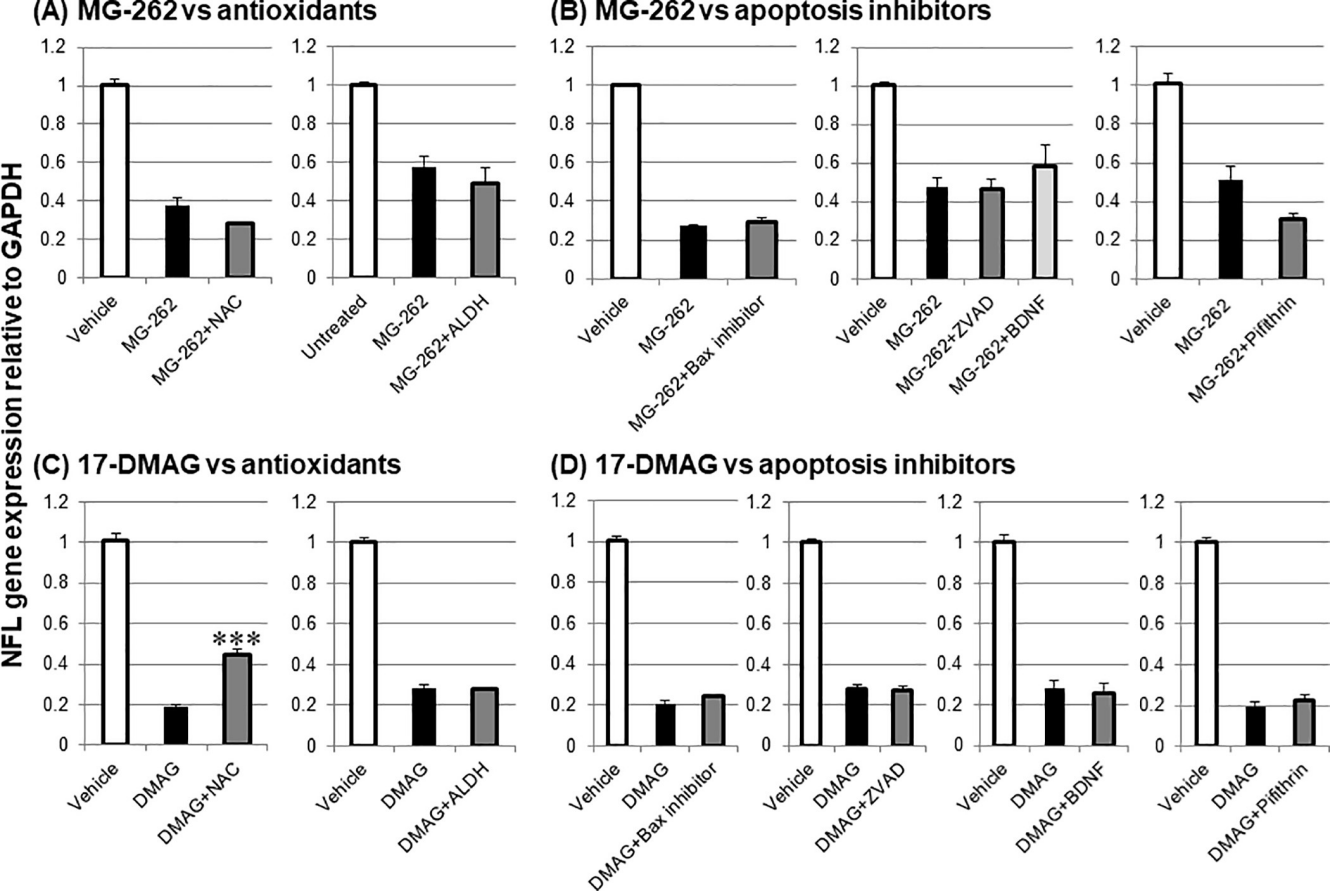
Modulatory effects of antioxidants and apoptosis inhibitors on downregulation of neurofilament light chain (NFL) gene expression 3 and 1 day following intravitreal injection of MG-262 and 17-DMAG, respectively. (A) Vehicle (10% DMSO in distilled water, open column), untreated (open column), MG-262 alone (closed column, 0.1 nmol/eye), and combined with N-acetyl cysteine (NAC, dark grey, 500 nmol/eye) or aldehyde dehydrogenase (ALDH, dark grey, 0.025 u/eye). (B) Vehicle (open column, 10–50% DMSO in distilled water), MG-262 alone (closed column, 0.1 nmol/eye) and combined with a bax channel blocker (bax inhibitor, dark grey, 25 nmol/eye), the caspase inhibitor Z-VAD-FMK (ZVAD, dark grey, 7.5 nmol/eye), brain derived neurotrophic factor (BDNF, light grey, 2 μg/eye) or the p53 inhibitor pifithrin-α, cyclic (pifithrin, dark grey, 1 nmol/eye). (C) Vehicle (open column, 20–30% DMSO in distilled water), 17-DMAG alone (closed column, DMAG, 25 nmol/eye) and combined with N-acetyl cysteine (NAC, dark grey, 500 nmol/eye) or aldehyde dehydrogenase (ALDH, dark grey, 0.025 u/eye). (D) Vehicle (open column, 20–33% DMSO in distilled water), 17-DMAG alone (closed column, DMAG, 25 nmol/eye) and combined with a bax channel blocker (bax inhibitor, dark grey, 25 nmol/eye), the caspase inhibitor Z-VAD-FMK (ZVAD, dark grey, 7.5 nmol/eye), brain derived neurotrophic factor (BDNF, dark grey, 2 μg/eye) or the p53 inhibitor pifithrin-α, cyclic (pifithrin, dark grey, 1 nmol/eye). Each pharmacological agent was premixed with a proteasome inhibitor and concurrently administered to animals. Following intravitreal injection, the retina was isolated and NFL gene expression was determined by real time PCR. The NFL gene expression level was normalized to that of GAPDH in each retinal sample and shown as the value relative to the respective control. Each value represents the mean ± S.E.M. of 4 to 8 eyes from 2 to 4 animals. ***P<0.001, compared with DMAG alone by Tukey’s multiple comparison test. Note that NFL downregulation by MG-262 and DMAG alone was statistically significant compared with the respective control group by Tukey’s multiple comparison test.

With the aid of this RGC marker, we first examined the effects of antioxidants and apoptosis inhibitors on MG-262 and 17-DMAG-induced RGC loss (Fig 9A–9D). Concomitant intravitreal injection of either NAC (500 nmol/eye) or ALDH (0.025 U/eye) with MG-262 (0.1 nmol/eye) had no effect on downregulated NFL gene expression by MG-262 alone (Fig 9A). In contrast, co-administration of NAC with 17-DMAG led to significantly less NFL gene downregulation than that seen with 17-DMAG injection alone, whereas a combination of ALDH with 17-DMAG had no effect (Fig 9C). None of a bax channel inhibitor (25 nmol/eye), the caspase inhibitor ZVAD (7.5 nmol/eye), BDNF (2 μg/eye), or the p53 inhibitor pifithrin (1 nmol/eye) affected MG-262- or 17-DMAG-induced NFL gene downregulation (Fig 9B and 9D). These results suggest that oxidative stress, but not apoptotic signaling, plays some roles in 17-DMAG-induced RGC loss, whereas either pathway does not contribute to MG-262-induced one.

**Fig 10:**
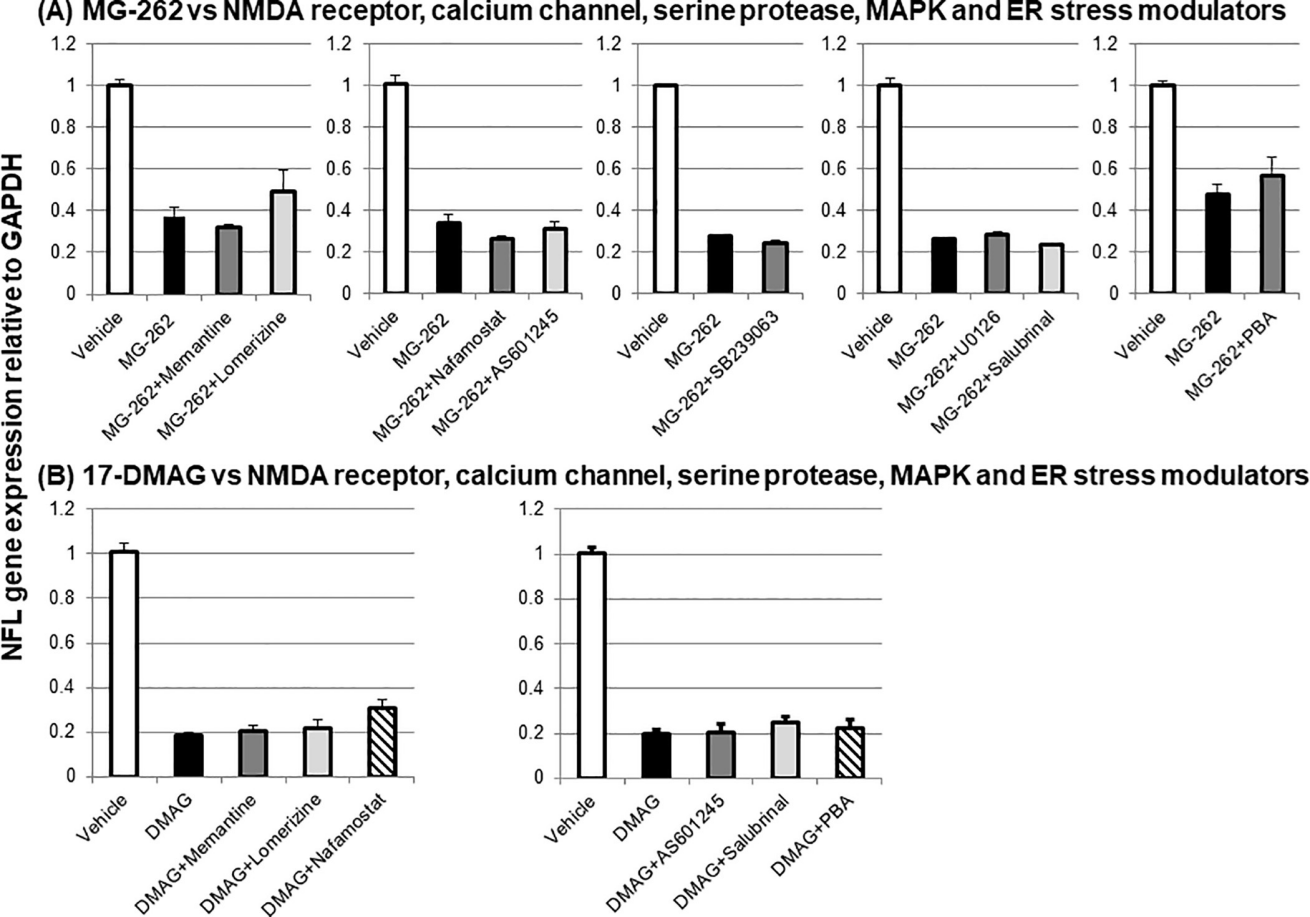
Effects of various pharmacological agents on downregulation of neurofilament light chain (NFL) gene expression 3 and 1 day following intravitreal injection of MG-262 and 17-DMAG, respectively. (A) Vehicle (open column, 10–100% DMSO in distilled water), MG-262 alone (closed column, 0.1 nmol/eye) and combined with the NMDA receptor antagonist memantine (dark grey, 100 nmol/eye), the calcium channel blocker lomerizine (light grey, 50 nmol/eye), the serine protease inhibitor nafamostat (dark grey, 10 nmol/eye), the JNK inhibitor AS601245 (light grey, 10.5 nmol/eye), the p38 inhibitor SB239063 (dark grey, 20 nmol/eye), the ERK inhibitor U0126 (dark grey, 50 nmol/eye), the ER stress modulator salubrinal (light grey, 50 nmol/eye) or sodium 4-phenyl butyrate (PBA, dark grey, 100 nmol/eye). (B) Vehicle (open column, 30–33% DMSO in distilled water), 17-DMAG alone (closed column, DMAG, 25 nmol/eye) and combined with memantine (dark grey, 100 nmol/eye), lomerizine (light grey, 50 nmol/eye), nafamostat (hatched, 10 nmol/eye), AS601245 (dark grey, 10.5 nmol/eye), salubrinal (light grey, 12.5 nmol/eye) or PBA (hatched, 100 nmol/eye). Each pharmacological agent was premixed with a proteasome inhibitor and concurrently administered to animals. Following intravitreal injection, the retina was isolated and NFL gene expression was determined by real time PCR. The NFL gene expression level was normalized to that of GAPDH in each retinal sample and shown as the value relative to the respective control. Each value represents the mean ± S.E.M. of 5 to 10 eyes from 3 to 5 animals. Note that NFL downregulation by MG-262 and DMAG alone was statistically significant compared with the respective control group by Tukey’s multiple comparison test.

The molecular mechanisms for chemical proteasome inhibition-induced retinal degeneration were further characterized using the NMDA antagonist memantine (100 nmol/eye), the calcium channel blocker lomerizine (50 nmol/eye), the serine protease inhibitor nafamostat (10 nmol/eye), MAP kinase inhibitors (the JNK inhibitor AS601245, 10.5 nmol/eye; the p38 inhibitor SB239063, 20 nmol/eye; the ERK inhibitor U0126, 50 nmol/eye), and the ER stress inhibitor salubrinal (12.5 or 50 nmol/eye) and PBA (100 nmol/eye). As shown in Fig 10, any of tested agents did not change either MG-262- or 17-DMAG-induced NFL gene downregulation (Fig 10A and 10B). Furthermore, MG-262-induced NFL gene downregulation was not modified by any other classes of tested agents (see S4 Fig), namely, ion transport modulators (the Na-K-Cl transport inhibitor bumetanide, 50 nmol/eye; the calmodulin inhibitor trifluoperazine, 25 nmol/eye; the calcium chelator BAPTA, 125 nmol/eye; the ion chelator deferoxamine, 100 nmol/eye; the Na/Ca exchanger blocker KB-R7943, 50 nmol/eye), unfolded-protein response (UPR) modulators (the GSK-3β inhibitor SB-216763, 0.085 nmol/eye; TWS119, 0.075 nmol/eye; the XBP-1 inhibitor ansatrienin A, 1 nmol/eye), protein synthesis modulators (the protein synthesis inhibitor cycloheximide, 10 nmol/eye; the protein-nucleic acid complex inhibitor aurintricarboxylic acid, 5 nmol/eye), and a protein aggregation inhibitor (C2-8, 1 nmol/eye). In all experiments, downregulation of NFL gene expression by MG-262 and 17-DMAG alone was statistically significant.

## Discussion

Our main finding in the present study is that intravitreal injection of the potent and selective proteasome inhibitor MG-262 [16] led to time- and dose-dependent inner retinal degeneration accompanied by reduced proteasome activity and poly-ubiquitinated protein accumulation in the normal adult rat retina. The most prominent feature of MG-262-induced inner retinal degeneration was cell loss in GCL accompanied by downregulation of NFL gene expression. Two structurally different proteasome inhibitors, lactacystin [17] and bortezomib [21], caused exactly the same pattern of inner retinal degeneration as MG-262 did. Furthermore, we found that 17-DMAG, a presumed HSP90 inhibitor, also suppressed proteasome activity and caused the same pattern of inner retinal degeneration, suggesting that 17-DMAG behaves as a direct/indirect proteasome inhibitor under our experimental conditions. Interestingly, intravitreal injection of two authentic ER stress inducers, tunicamycin and thapsigargin [18], resulted in massive retinal degeneration predominantly in the photoreceptor layer, which was a distinguishable pattern from that observed with chemical proteasome inhibitors. To our best knowledge, this is the first report demonstrating the unique pattern of inner retinal degeneration induced by chemical proteasome inhibitors. Furthermore, our study provides *in vivo* evidence to further support that the UPS may play a crucial role in the maintenance of the structural integrity of the normal adult retina.

A possible drawback of our pharmacological approaches in this study may be the specificity of each agent used, particularly, MG-262. To address this specificity issue, we carefully designed this study in the following ways: 1) to analyze dose-dependency of its retinal degenerative effect, 2) to cross-check the results with use of four structurally different proteasome inhibitors (including 17-DMAG), and 3) to obtain direct evidence of involvement of reduced proteasome activity in retinal degeneration. All the data obtained in this study were consistent: The retinal degenerative effects of MG-262 and 17-DMAG were dose-dependent; All the proteasome inhibitors caused the same pattern of inner retinal degeneration; MG-262 reduced proteasome activity, and increased poly-ubiquitinated protein levels and ubiquitin-positive immunostaining in the retina. However, one might still argue that the used concentrations of proteasome inhibitors would not have been either sufficient or selective for proteasome inhibition. The concentrations of MG-262 (0.01–0.1 nmol/eye) in the vitreous humor were estimated to be 0.17–1.7 μM, assuming that the vitreous volume of the rat eye is 60 μl [26]. In this concentration range, MG-262 completely inhibited proteasome activity in our cell-free assay, and these concentrations are comparable to the *in vitro* concentrations used in earlier studies [27, 28]. The estimated vitreous concentrations of lactacystin (10 nmol/eye) and 17-DMAG (25 nmol/eye) are 170 and 425 μM, respectively, which are also close to their maximum inhibitory concentrations in our cell-free assay. The estimated bortezomib vitreous concentration (1 nmol/eye) is 17 μM and relatively higher than its maximum inhibitory concentrations in the same assay. Generally, the *in vivo* activities of any chemicals are highly affected by their retinal distribution and cell penetration following intravitreal injection [29], which may limit intracellular concentrations in target cells such as RGCs. Therefore, in our *in vivo* setting, the used concentrations of each proteasome inhibitor seem to be reasonable for sufficient and selective inhibition of retinal proteasome activity.

The primary contribution of this study is to provide a novel animal model of inner retinal degeneration through chemical proteasome inhibition by single intravitreal injection. Even though chemical proteasome inhibition has been established and extensively characterized as an animal model for Parkinson’s disease [10], little attention has been paid to its potential application to the retina. An attempt was made to examine the retinal effect of bortezomib administered intraperitoneally to rats [30]. However, this earlier study was focused on the retinal protective effect of bortezomib against ischemic injury, but not on its own effect on the normal retinal morphology or function. More relevantly, the retinal toxic effect of 17-DMAG following repeated intravenous administration was reported in rats [31]. The authors proposed that systemic administration of 17-DMAG led exclusively to photoreceptor damage (ONL and IS/OS), which might be associated with its clinical adverse events including blurred vision [32, 33]. This finding is inconsistent with our current result that intravitreal injection of 17-DMAG caused inner retinal degeneration. This inconsistency may be due to differences in the route of administration and dosing regimens. For instance, systemic administration delivers 17-DMAG mainly to the outer retina through the blood-retinal barrier, whereas intravitreal administration delivers it directly to the inner retina. Because of its hydrophilicity [29], there might be a possibility that this compound may be retained mainly in the outer and inner retina following systemic and intravitreal administration, respectively, resulting in retinal degeneration at each location. In contrast, MG-262 and bortezomib with much more lipophilic nature may readily diffuse and distribute throughout the retina [29], but they caused mainly inner retinal degeneration. Furthermore, tunicamycin and thapsigargin, other lipophilic agents, caused preferential outer retinal degeneration, but did not affect the inner retina. Therefore, it is most likely that inner retinal degeneration induced by lipophilic proteasome inhibitors may be due to vulnerability of inner retinal cells including RGC to chemical proteasome inhibition, but not due to their physicochemical or pharmacokinetic properties.

Genetic ablation of proteasome function is an alternative way to make an animal model of proteasome inhibition-induced retinal degeneration. Two earlier studies using this approach reported that 20S proteasome inhibition by β5t transgene [6] and immunoproteasome inhibition by lmp7 and mecl-1 double knockout [34] caused photoreceptor cell degeneration and impaired bipolar cell function, respectively. Surprisingly, no changes in RGCs were observed in either study. The exact mechanism for this discrepancy is not clear, but it may be attributed to differences in the retinal localization of targeted proteins and/or modes of action of proteasome inhibition, i.e., transgenic expression of an β5-like inactive form to replace native β5 subunits in the 20S proteasome complex [6], genetic deletions of catalytic subunits of the immunoproteasome [34], and binding of a chemical inhibitor to the catalytic active site of the β5 subunit [9]. Nevertheless, the most important technical advantage of our chemical model over these genetic models is that dosing regimens of chemical proteasome inhibitors determine the degree of severity and timing of inner retinal degeneration, which can be optimized depending on research objectives. Furthermore, rapid and reproducible retinal degeneration can be achieved simply by intravitreal injection of small chemicals. Such advantages minimize time and costs to establish this animal model and further evaluate therapeutic compounds. Taken together, in-depth characterization of this unique model may provide further insights into the underlying molecular mechanisms for inner retinal degeneration associated with misfolded proteins and may eventually lead to discovery of novel therapeutic targets.

Another aspect of the current study is to examine how each component of protein degradation processes in the UPS would contribute to the maintenance of the retinal structural integrity. To address this point, we used specific chemical inhibitors for the following steps: 1) brefeldin A for protein translocation from ER to the Golgi apparatus [23], 2) 17-DMAG for protein folding through binding to the chaperon protein HSP90 [22], 3) UBEI-41 for ubiquitination [19], 4) MG-262, lactacystin and bortezomib for 20S proteasomal proteolysis [35], and 5) LDN-57444 for deubiquitination [20]. Consequently, we found that intravitreal injection of HSP90 and 20S proteasome inhibitors caused inner retinal degeneration, whereas inhibition of the rest of steps did not. Interestingly, we demonstrated that the HSP90 inhibitor 17-DMAG reduced 20S proteasome activity even in a cell-free assay using a purified enzyme, suggesting the role for proteasome inhibition in its retinal degenerative effect. Earlier studies reported that HSP90 formed a complex with 20S proteasome, which promoted degradation of an oxidized protein [36] and processing of major histocompatibility complex class I antigen [37]. Likewise, it is reasonable to speculate that inhibition by 17-DMAG of proteasome activity in our cell-free assay may be due to a purified 20S proteasome enzyme assembled with HSP90, which may more likely happen *in vivo*. Thus, our study indicates that 20S proteasome is the primary component of the UPS in the maintenance of the retinal structural integrity through controlling retinal protein degradation.

In this study, we notice that the degree of reduced proteasome activity (30%) is not quantitatively consistent with that of reduced cell number in GCL (70%) following intravitreal injection of MG-262 at the dose of 0.1 nmol/eye. This is because the time points for these two measurements were different; ex vivo proteasome activity was measured 3 days following the injection, whereas the cell number was counted 7 days following the injection. A similar finding was reported in an earlier study using β5t transgenic mice[6]; Proteasome activity was reduced by approximately 30% at the age of 1 month of transgenic mice, whereas no retinal morphological changes were observed at the same age. However, ONL thickness was reduced by approximately 60% at the age of 9 months. Thus, reduced proteasome activity may proceed retinal morphological changes and these parameters may be correlated with each other qualitatively, but not quantitatively. Further studies are underway to address this discrepancy between temporal profiles of proteasome activity and morphological changes following chemical proteasome inhibition.

It is of great interest in this study to elucidate the molecular mechanisms underlying inner retinal degeneration induced by chemical proteasome inhibition. We first hypothesized that like ER stress inducers [38, 39], chemical proteasome inhibition would provoke the UPR including upregulation of CHOP [40], leading to apoptosis of retinal cells. This hypothesis was apparently supported by the finding that MG-262 increased retinal CHOP-positive immunostaining. However, we could not believe that the mechanisms of action of ER stress inducers and proteasome inhibitors would be identical, given the fact that chemical proteasome inhibition caused mainly inner retinal degeneration, whereas ER stress inducers caused exclusive outer retinal degeneration, which is consistent with the earlier findings [41, 42]. In fact, our extensive pharmacological approaches demonstrated that either MG-262 or 17-DMAG-induced retinal degeneration was not prevented by any of inhibitors of ER stress, apoptosis or MAP kinase signaling associated with the UPR [40]. Our finding is supported by the results of recent studies demonstrating that genetic ablation of CHOP did not prevent photoreceptor death in rhodopsin mutant mice [43–45], although CHOP knockout was reported to partially protect RGCs against cell death induced by NMDA [46], retinal ischemia [47], and optic nerve crush [48]. These results suggest that relative contribution of the UPR to retinal cell death varies depending on types of retinal injury, and that it plays a minimal role in retinal cell death caused by chemical proteasome inhibition and misfolded mutant proteins. Alternatively, glutamate toxicity [49], ion imbalance, and oxidative stress [10] would be the possible mechanisms underlying retinal degeneration induced by chemical proteasome inhibition. Among them, only oxidative stress may play some role in DMAG-induced retinal degeneration, because 17-DMAG-induced NFL gene downregulation was partially suppressed by the antioxidant NAC, but not by any of a glutamate receptor antagonist or ion transporter modulators. This result is in line with those in earlier studies demonstrating that oxidative stress contributes to neuronal cell death induced by lactacystin and epoxomicin in PC12 cells [50] and mesencephalic neurons [51], respectively. However, any pharmacological agents did not modify MG-262-induced retinal degeneration, suggesting that the signaling pathways associated with retinal degeneration are different between direct 20S proteasome inhibition by MG-262 and indirect proteasome inhibition through interaction of 17-DMAG with HPS90. Thus, other unidentified mechanisms may play major roles in chemical proteasome inhibition-induced inner retinal degeneration.

In conclusion, the present study provides a unique and versatile animal model of inner retinal degeneration, including RGC loss, following intravitreal injection of chemical proteasome inhibitors. We also demonstrate indispensable roles for the UPS in the maintenance of the structural integrity of the normal adult retina. At present, we can only suggest that morphological and biochemical features of our animal model replicate some of clinical features of inner retinal diseases such as glaucoma and diabetic retinopathy, because accumulating evidence supports the roles for impaired UPS in multiple retinal diseases as reviewed by Campello et al [52]. Further studies are underway to gain more insights into the exact molecular mechanisms underlying chemical proteasome inhibition-induced inner retinal degeneration. Such an attempt may lead to identification of novel therapeutic targets for neurodegenerative diseases associated with misfolded proteins.

## Supporting information

S1 Fig MG-262-induced retinal cell degeneration in the adult rat eyes. Transmission electron microscopic images of the normal adult rat retina exposed to either vehicle (A, 50% DMSO in D-PBS) or MG-262 (B, 0.1 nmol/eye). Twenty-four hours following intravitreal injection, the eyes were isolated and ultra-thin sections were prepared. The left images show the structure of cells in the outer nuclear layer (ONL) and the inner segment (IS), whereas the right ones show that in the inner nuclear layer (INL). Nuclear condensation of photoreceptors in ONL and necrosis of bipolar cell in INL were observed only following MG-262 injection (arrows). The scale bar shows 2 μm.
(TIF)

S2 Fig No effects of LDN-57444 and brefeldin A on the retinal morphology in the adult rat eyes. Vehicle (10% DMSO in D-PBS), LDN-57444 (2.5 nmol/eye) or brefeldin A (1.5 nmol/eye) was injected into the vitreous body of the normal adult rat eyes. (A) and (B) show the number of cells in the ganglion cell layer (GCL) and the thickness of the inner plexiform layer (IPL), respectively. Each value represents the mean ± S.E.M. of 5 to 6 eyes from 3 animals. The values in groups treated with each chemical were not statistically different from that in the vehicle-treated group.
(TIF)

S3 Fig Retinal poly-ubiquitinated protein levels following intravitreal injection of MG-262. MG-262 (closed columns) was administered at the dose of 0.03 nmol/eye into the vitreous body of the normal adult rat eyes. For the control group (open column), vehicle (10% DMSO in distilled water) was injected. Three days following intravitreal injection, the retina was isolated and poly-ubiquitinated protein levels in each retinal lysate were determined by ELISA. The retinal poly-ubiquitinated protein level was normalized to a total protein content in each retinal lysate. Each value represents the mean ± S.E.M. of 4 eyes from 2 animals. No statistically significant change was observed between the groups.
(TIF)

S4 Fig No effects of various pharmacological agents on downregulation of neurofilament light chain (NFL) gene expression following intravitreal injection of MG-262 in the normal adult rat retina. (A-F) Vehicle (open column, 10–100% DMSO in distilled water) and MG-262 alone (black column, 0.1 nmol/eye). MG-262 was co-administered with: (A) Na-K-Cl transport inhibitor bumetanide (dark grey, 50 nmol/eye), the calmodulin inhibitor trifluoperazine (light grey, 25 nmol/eye) or the calcium chelator BAPTA (hatched, 125 nmol/eye); (B) the ion chelator deferoxamine (dark grey, 100 nmol/eye); (C) the Na/Ca exchanger blocker KB-R7943 (dark grey, 50 nmol/eye); (D) the GSK-3β inhibitor SB-216763 (dark gray 0.085 nmol/eye) or TWS119 (light gray, 0.075 nmol/eye); (E) the XBP-1 inhibitor ansatrienin A (dark grey, 1 nmol/eye), the protein synthesis inhibitor cycloheximide (light grey, 10 nmol/eye) or the protein aggregation inhibitor C2-8 (C2-8, hatched, 1 nmol/eye); (F) the protein-nucleic acid complex inhibitor aurintricarboxylic acid (ATA, dark grey, 5 nmol/eye). Each pharmacological agent was premixed and concurrently administered with MG-262 into the vitreous body of the normal adult rat eyes. One day (E) or three days (A, B, C, D, and F) following intravitreal injection, the retina was isolated and NFL gene expression was determined by real time PCR. The NFL gene expression level was normalized to that of GAPDH in each retinal sample and shown as the value relative to the respective control. Each value represents the mean ± S.E.M. of 1 to 8 eyes from 1 to 4 animals. No statistically significant change was observed between groups treated with each pharmacological agent and MG-262 alone. Note that NFL downregulation by MG-262 alone was statistically significant compared with the respective control group by Tukey’s multiple comparison test.
(TIF)

S1 Table Semi-quantitative measurements of ubiquitin, 20S proteasome and GADD153/CHOP-positive immunostaining following intravitreal injection of MG-262 in the normal adult rat retina. One, six and twenty-four hours following intravitreal injection of vehicle (A, 50% DMSO in distilled water) and MG-262 (B, 0.1 nmol/eye), eyes were enucleated and the retina was subjected to immunohistochemical staining using antibodies against ubiquitin (S1A), 20S proteasome subunit (S1B) and GADD153/CHOP (S1C). The intensity of each signal was scored as 0: negative; 1: slightly positive; 2: moderately; 3: strongly. NFL: nerve fiber layer; GCL: ganglion cell layer; IPL: inner plexiform layer; INL: inner nuclear layer; OPL: outer plexiform layer; ONL: outer nuclear layer; IS/OS: inner/outer segments; RPE: retinal pigment epithelium.
(DOCX)
