# Supplementary figures and images for: Chemical proteasome inhibition as a novel animal model of inner retinal degeneration in rats
Source: PLoS One. 2019 May 31;14(5):e0217945. doi: 10.1371/journal.pone.0217945 (PMC6544319; doi:10.1371/journal.pone.0217945)

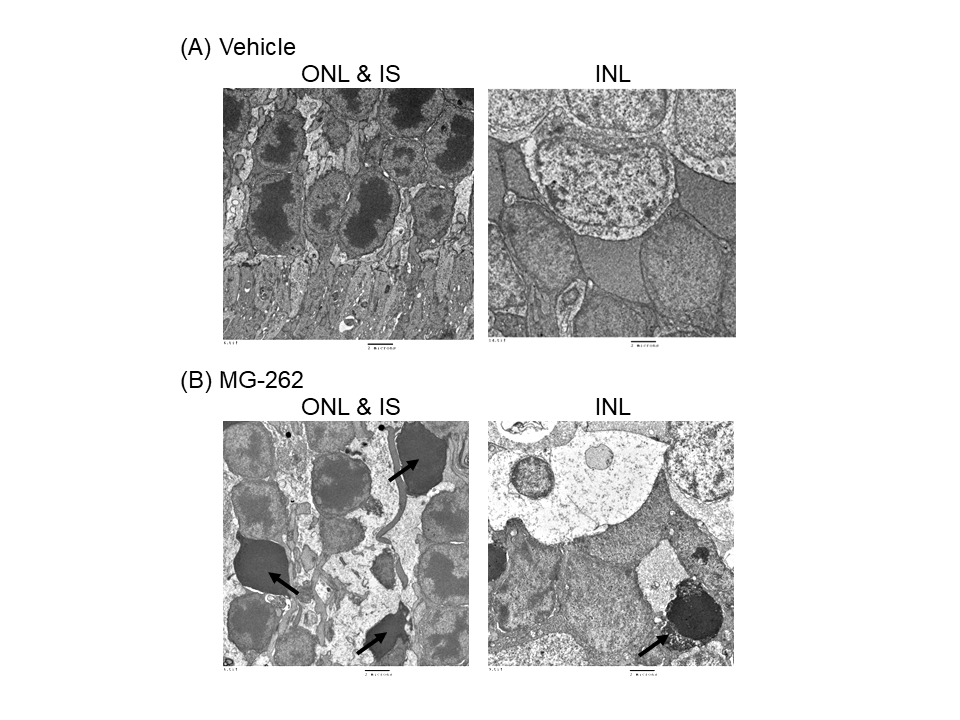

Supplement: S1 Fig — Transmission electron microscopic images of the normal adult rat retina exposed to either vehicle (A, 50% DMSO in D-PBS) or MG-262 (B, 0.1 nmol/eye). Twenty-four hours following intravitreal injection, the eyes were isolated and ultra-thin sections were prepared. The left images show the structure of cells in the outer nuclear layer (ONL) and the inner segment (IS), whereas the right ones show that in the inner nuclear layer (INL). Nuclear condensation of photoreceptors in ONL and necrosis of bipolar cell in INL were observed only following MG-262 injection (arrows). The scale bar shows 2 μm. (TIF) [file pone.0217945.s001.TIF]

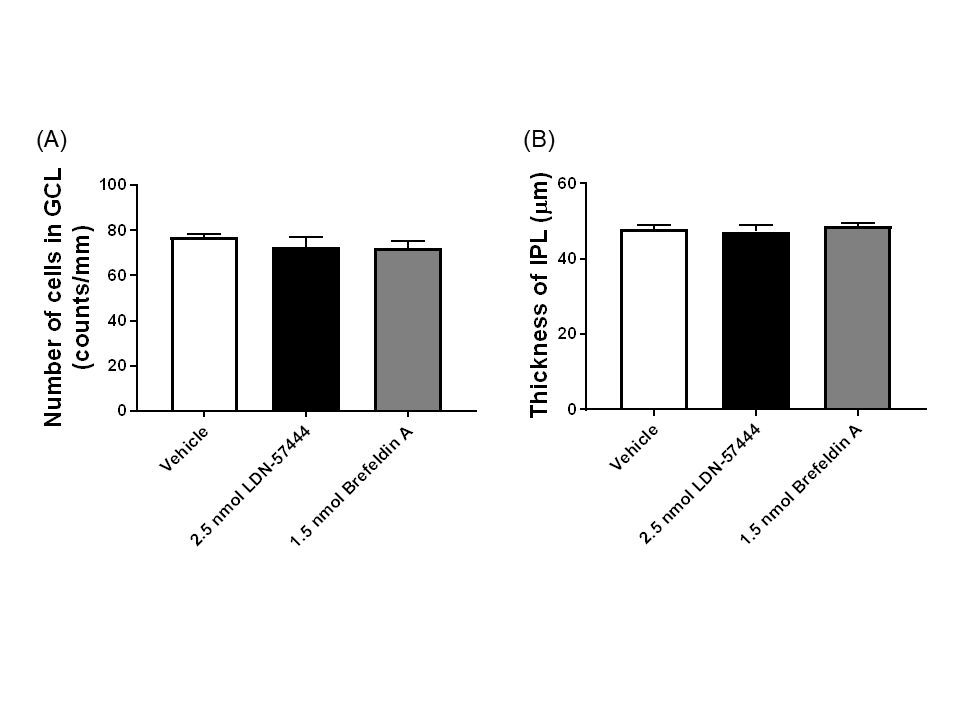

Supplement: S2 Fig — Vehicle (10% DMSO in D-PBS), LDN-57444 (2.5 nmol/eye) or brefeldin A (1.5 nmol/eye) was injected into the vitreous body of the normal adult rat eyes. (A) and (B) show the number of cells in the ganglion cell layer (GCL) and the thickness of the inner plexiform layer (IPL), respectively. Each value represents the mean ± S.E.M. of 5 to 6 eyes from 3 animals. The values in groups treated with each chemical were not statistically different from that in the vehicle-treated group. (TIF) [file pone.0217945.s002.TIF]

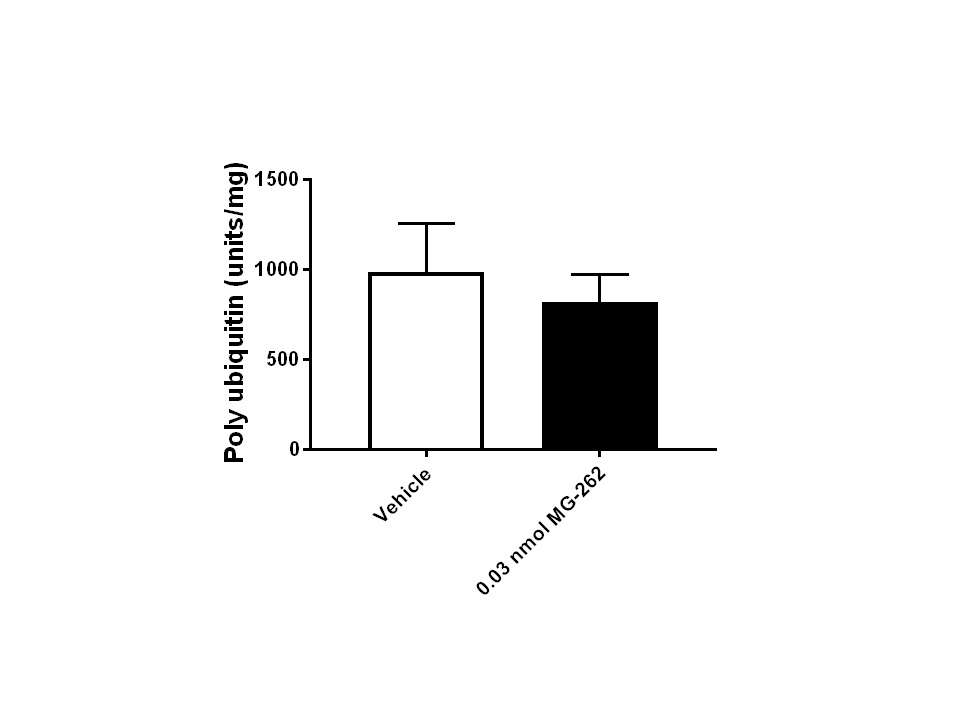

Supplement: S3 Fig — MG-262 (closed columns) was administered at the dose of 0.03 nmol/eye into the vitreous body of the normal adult rat eyes. For the control group (open column), vehicle (10% DMSO in distilled water) was injected. Three days following intravitreal injection, the retina was isolated and poly-ubiquitinated protein levels in each retinal lysate were determined by ELISA. The retinal poly-ubiquitinated protein level was normalized to a total protein content in each retinal lysate. Each value represents the mean ± S.E.M. of 4 eyes from 2 animals. No statistically significant change was observed between the groups. (TIF) [file pone.0217945.s003.TIF]

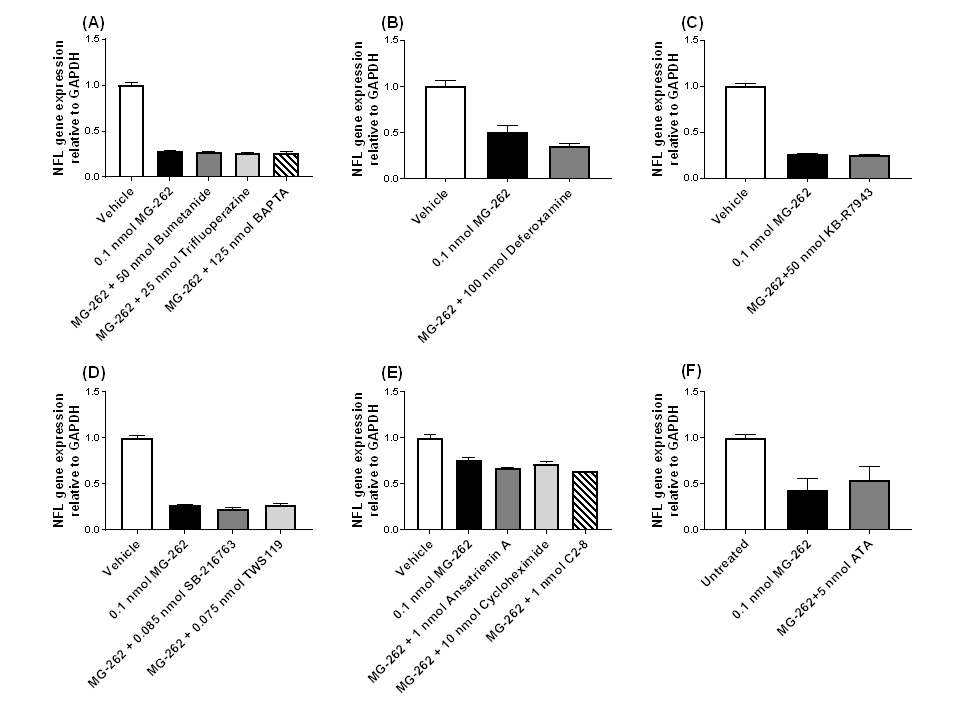

Supplement: S4 Fig — (A-F) Vehicle (open column, 10–100% DMSO in distilled water) and MG-262 alone (black column, 0.1 nmol/eye). MG-262 was co-administered with: (A) Na-K-Cl transport inhibitor bumetanide (dark grey, 50 nmol/eye), the calmodulin inhibitor trifluoperazine (light grey, 25 nmol/eye) or the calcium chelator BAPTA (hatched, 125 nmol/eye); (B) the ion chelator deferoxamine (dark grey, 100 nmol/eye); (C) the Na/Ca exchanger blocker KB-R7943 (dark grey, 50 nmol/eye); (D) the GSK-3β inhibitor SB-216763 (dark gray 0.085 nmol/eye) or TWS119 (light gray, 0.075 nmol/eye); (E) the XBP-1 inhibitor ansatrienin A (dark grey, 1 nmol/eye), the protein synthesis inhibitor cycloheximide (light grey, 10 nmol/eye) or the protein aggregation inhibitor C2-8 (C2-8, hatched, 1 nmol/eye); (F) the protein-nucleic acid complex inhibitor aurintricarboxylic acid (ATA, dark grey, 5 nmol/eye). Each pharmacological agent was premixed and concurrently administered with MG-262 into the vitreous body of the normal adult rat eyes. One day (E) or three days (A, B, C, D, and F) following intravitreal injection, the retina was isolated and NFL gene expression was determined by real time PCR. The NFL gene expression level was normalized to that of GAPDH in each retinal sample and shown as the value relative to the respective control. Each value represents the mean ± S.E.M. of 1 to 8 eyes from 1 to 4 animals. No statistically significant change was observed between groups treated with each pharmacological agent and MG-262 alone. Note that NFL downregulation by MG-262 alone was statistically significant compared with the respective control group by Tukey’s multiple comparison test. (TIF) [file pone.0217945.s004.tif]
