# Supplementary material for: Chemical proteasome inhibition as a novel animal model of inner retinal degeneration in rats
Source: PLoS One. 2019 May 31;14(5):e0217945. doi: 10.1371/journal.pone.0217945 (PMC6544319; doi:10.1371/journal.pone.0217945)
Supplement: S1 Table — One, six and twenty-four hours following intravitreal injection of vehicle (A, 50% DMSO in distilled water) and MG-262 (B, 0.1 nmol/eye), eyes were enucleated and the retina was subjected to immunohistochemical staining using antibodies against ubiquitin (S1A), 20S proteasome subunit (S1B) and GADD153/CHOP (S1C). The intensity of each signal was scored as 0: negative; 1: slightly positive; 2: moderately; 3: strongly. NFL: nerve fiber layer; GCL: ganglion cell layer; IPL: inner plexiform layer; INL: inner nuclear layer; OPL: outer plexiform layer; ONL: outer nuclear layer; IS/OS: inner/outer segments; RPE: retinal pigment epithelium. (DOCX) [file pone.0217945.s005.docx]

**S1A Table. Ubiquitin-positive immunostaining.**

| **Vehicle** | **Sample ID** | **NFL** | **GCL** | **IPL** | **INL** | **OPL** | **ONL** | **IS/OS** | **RPE** |
| --- | --- | --- | --- | --- | --- | --- | --- | --- | --- |
| 1 hr | 001* | 2 | 0 | 1 | 0 | 1 | 1 | 1 | 1 |
|  | 002 | 2 | 0 | 2 | 0 | 1 | 1 | 1 | 0 |
| 6 hr | 003 | 2 | 0 | 1 | 0 | 1 | 1 | 1 | 0 |
|  | 004 | 2 | 0 | 1 | 0 | 1 | 1 | 1 | 1 |
| 24 hr | 005 | 2 | 0 | 1 | 0 | 1 | 1 | 1 | 0 |
|  | 006 | 2 | 0 | 2 | 0 | 1 | 1 | 1 | 0 |
|  | | | | | | | | | |
| **MG-262** | **Sample ID** | **NFL** | **GCL** | **IPL** | **INL** | **OPL** | **ONL** | **IS/OS** | **RPE** |
| 1 hr | 007 | 2 | 0 | 2 | 0 | 1 | 1 | 1 | 0 |
|  | 008 | 2 | 0 | 2 | 0 | 1 | 1 | 1 | 1 |
| 6 hr | 009 | 2 | 0 | 2 | 0 | 1 | 1 | 1 | 1 |
|  | 010 | 2 | 0 | 2 | 0 | 1 | 1 | 1 | 1 |
| 24 hr | 011 | 2 | 0 | 3 | 1 | 2 | 2 | 1 | 1 |
|  | 012^#^ | 2 | 0 | 3 | 2 | 2 | 2 | 1 | 1 |

**S1B Table. 20S proteasome-positive immunostaining.**

| **Vehicle** | **Sample ID** | **NFL** | **GCL** | **IPL** | **INL** | **OPL** | **ONL** | **IS/OS** | **RPE** |
| --- | --- | --- | --- | --- | --- | --- | --- | --- | --- |
| 1 hr | 001* | 1 | 0 | 0 | 0 | 1 | 1 | 1 | 0 |
|  | 002 | 2 | 0 | 1 | 0 | 2 | 1 | 1 | 1 |
| 6 hr | 003 | 1 | 0 | 1 | 0 | 1 | 1 | 1 | 1 |
|  | 004 | 2 | 0 | 1 | 0 | 1 | 1 | 1 | 1 |
| 24 hr | 005 | 2 | 0 | 1 | 0 | 1 | 1 | 1 | 1 |
|  | 006 | 2 | 0 | 1 | 0 | 1 | 1 | 1 | 1 |
|  | | | | | | | | | |
| **MG-262** | **Sample ID** | **NFL** | **GCL** | **IPL** | **INL** | **OPL** | **ONL** | **IS/OS** | **RPE** |
| 1 hr | 007 | 2 | 0 | 1 | 0 | 2 | 1 | 1 | 0 |
|  | 008 | 2 | 0 | 1 | 0 | 2 | 1 | 1 | 1 |
| 6 hr | 009 | 2 | 0 | 0 | 0 | 1 | 1 | 1 | 0 |
|  | 010 | 2 | 0 | 1 | 0 | 1 | 1 | 1 | 0 |
| 24 hr | 011 | 2 | 0 | 1 | 1 | 2 | 1 | 1 | 1 |
|  | 012^#^ | 2 | 0 | 1 | 1 | 2 | 1 | 1 | 0 |

**S1C Table. GADD153/CHOP-positive immunostaining.**

| **Vehicle** | **Sample ID** | **NFL** | **GCL** | **IPL** | **INL** | **OPL** | **ONL** | **IS/OS** | **RPE** |
| --- | --- | --- | --- | --- | --- | --- | --- | --- | --- |
| 1 hr | 001* | 1 | 0 | 1 | 0 | 1 | 0 | 1 | 1 |
|  | 002 | 1 | 0 | 1 | 0 | 1 | 0 | 1 | 1 |
| 6 hr | 003 | 1 | 0 | 1 | 0 | 1 | 0 | 0 | 1 |
|  | 004 | 1 | 0 | 1 | 0 | 1 | 0 | 0 | 1 |
| 24 hr | 005 | 1 | 0 | 1 | 0 | 1 | 0 | 0 | 1 |
|  | 006 | 1 | 0 | 1 | 0 | 1 | 0 | 0 | 1 |
|  | | | | | | | | | |
| **MG-262** | **Sample ID** | **NFL** | **GCL** | **IPL** | **INL** | **OPL** | **ONL** | **IS/OS** | **RPE** |
| 1 hr | 007 | 1 | 1 | 1 | 0 | 1 | 0 | 1 | 1 |
|  | 008 | 1 | 1 | 1 | 0 | 1 | 0 | 1 | 1 |
| 6 hr | 009 | 1 | 1 | 1 | 0 | 1 | 0 | 1 | 1 |
|  | 010 | 1 | 1 | 1 | 0 | 1 | 0 | 0 | 1 |
| 24 hr | 011^#^ | 1 | 2 | 1 | 1 | 1 | 0 | 0 | 1 |
|  | 012 | 1 | 1 | 1 | 1 | 1 | 0 | 0 | 1 |

*: Images of the retina exposed to vehicle alone depicted in Fig. 7.

^#^: Images of the retina exposed to MG-262 depicted in Fig. 7.
